# Generation of a Mouse Model with Down-Regulated U50 snoRNA (SNORD50) Expression and Its Organ-Specific Phenotypic Modulation

**DOI:** 10.1371/journal.pone.0072105

**Published:** 2013-08-26

**Authors:** Yuuichi Soeno, Kazuya Fujita, Tomoo Kudo, Masataka Asagiri, Shigeru Kakuta, Yuji Taya, Yoshihito Shimazu, Kaori Sato, Ritsuko Tanaka-Fujita, Sachiko Kubo, Yoichiro Iwakura, Yoshikazu Nakamura, Shigeo Mori, Takaaki Aoba

**Affiliations:** 1 Department of Pathology, School of Life Dentistry, The Nippon Dental University, Tokyo, Japan; 2 Department of Pathology, Hyogo College of Medicine, Hyogo, Japan; 3 Innovation Center for Immunoregulation and Therapeutics, Graduate School of Medicine, Kyoto University, Kyoto, Japan; 4 Department of Biomedical Science, Graduate School of Agricultural and Life Sciences, University of Tokyo, Tokyo, Japan; 5 Center for Experimental Medicine and Systems Biology, Institute of Medical Science, University of Tokyo, Tokyo, Japan; 6 Risk Assessment Division, Food Safety Commission Secretariat, Cabinet Office, Government of Japan, Tokyo, Japan; 7 Division of Experimental Animal Immunology, Research Institute for Biomedical Sciences, Tokyo University of Science, Chiba, Japan; 8 Division of RNA Medical Science, Institute of Medical Science, University of Tokyo, Tokyo, Japan; 9 Ribomic Inc., Tokyo, Japan; 10 Kotobiken Medical Laboratories Inc., Tokyo, Japan; University of Münster, Germany

## Abstract

Box C/D-type small nucleolar RNAs (snoRNAs) are functional RNAs responsible for mediating 2′-*O*-ribose methylation of ribosomal RNAs (rRNAs) within the nucleolus. In the past years, evidence for the involvement of human U50 snoRNA in tumorigenesis has been accumulating. We previously identified *U50HG*, a non-protein-coding gene that hosted a box C/D-type U50 snoRNA, in a chromosomal breakpoint in a human B-cell lymphoma. Mouse genome analysis revealed four mouse U50 (mU50) host-genes: three *mU50HG-a* gene variants that were clustered in the genome and an *mU50HG-b* gene that we supposed to be the *U50HG* ortholog. In this study, to investigate the physiological importance of mU50 snoRNA and its involvement in tumorigenesis, we eliminated mU50 snoRNA sequences from the *mU50HG-b* gene. The established mouse line (ΔmU50_(HG-b)_) showed a significant reduction of mU50 snoRNA expression without alteration of the host-gene length and exon-intron structure, and the corresponding target rRNA methylation in various organs was reduced. Lifelong phenotypic monitoring showed that the ΔmU50_(HG-b)_ mice looked almost normal without accelerated tumorigenicity; however, a notable difference was the propensity for anomalies in the lymphoid organs. Transcriptome analysis showed that dozens of genes, including heat shock proteins, were differentially expressed in ΔmU50_(HG-b)_ mouse lymphocytes. This unique model of a single snoRNA knockdown with intact host-gene expression revealed further new insights into the discrete transcriptional regulation of multiple mU50 host-genes and the complicated dynamics involved in organ-specific processing and maintenance of snoRNAs.

## Introduction

Small nucleolar RNAs (snoRNAs) are one of the abundant non-protein-coding RNA species (>300 snoRNAs have been found in human) that are responsible for the maturation of ribosomal RNAs (rRNAs) within the nucleolus [1], [2]. snoRNAs achieve site-specific nucleotide modification by base-pairing to complementary sequences on rRNA precursors. Based on the conserved nucleotide sequences and their function, snoRNAs are grouped into two classes: box C/D-type snoRNAs and box H/ACA-type snoRNAs that catalyze 2′-*O*-ribose methylation and pseudouridylation, respectively [3]–[6]. Eukaryotic snoRNA genes are commonly harbored in introns of their host-genes, and snoRNA expression is coupled with splicing of the host-gene pre-mRNA [7]–[9], although some H/ACA-type snoRNAs have alternative routes of maturation [10]. Most snoRNA host-genes encode proteins that play a role in the translation machinery of cells. On the other hand, there are several snoRNA host-genes that do not encode proteins but instead possess a 5′-terminal oligopyrimidine (5′TOP) motif that is reminiscent of the signal sequence of ribosomal protein genes [11]–[13]. With respect to the physiological roles of snoRNAs, loss-of-function studies for a single snoRNA in yeast have so far demonstrated that the snoRNA-assisted rRNA modifications were not critical but, in part, caused subtle phenotypic alterations in cell growth [14], [15]. However, molecular genetic approaches have rarely been used to explore the physiological and pathogenetic importance of individual snoRNAs in higher organisms [16].

In a previous investigation of a novel translocation partner of *BCL6* at t(3;6)(q27;q15) in a human diffuse large B-cell lymphoma, we found a snoRNA host-gene, *U50HG*, that encoded U50 snoRNA within its intron [17] (*U50HG* and U50 snoRNA are now named *SNHG5* and *SNORD50*, respectively). U50 snoRNA belongs to the box C/D snoRNA class and has two regions in its sequence that are complementary to the human 28S rRNA [3], [4]. The U50 snoRNA is highly expressed in hematopoietic and lymphoid organs such as thymus, spleen, bone marrow, and lymph nodes [17]. *U50HG* has been identified as a non-protein-coding host-gene with a 5′TOP motif. Regarding the involvement of U50 snoRNA in human tumorigenicity, recent studies have reported that a genomic mutation in the U50 snoRNA gene (deletion of two thymidine residues in the middle of the gene) was related to poor prognosis in cancer patients [18], [19]. It has also been reported that over-expression of U50 snoRNA inhibited colony formation of human prostate cancer and breast cancer cell lines *in vitro*, suggesting that U50 snoRNA may behave as a tumor suppressor [18], [19]. At present, although details of the organ-specific regulation of *U50HG* and U50 snoRNA expression still remain elusive, it is an intriguing hypothesis that the perturbation of U50 snoRNA alone or coupled with an anomalous host-gene function might evoke causative or additive effects on tumorigenesis in an organ-specific manner.

During a genomic search for U50-related genes in mice, we previously identified a mouse U50 (mU50) snoRNA and the two 5′TOP non-protein-coding host-genes, *mU50HG-a* and *mU50HG-b* on mouse chromosome 9E3-F1 (syntenic with human 6q15 where *U50HG* is located) [20]. In that report, we proposed that *mU50HG-b* might be an ortholog to the human *U50HG* gene because of the structural similarity between the two genes and that *mU50HG-a* was presumably duplicated from *mU50HG-b* in mouse [20].

Based on these findings, we have generated a novel mouse model in which the expression of *mU50HG-b*-derived mU50 snoRNA is depleted without altering the host-gene length and genomic structure. Using this mouse model, it should be possible to investigate the physiological roles of the single snoRNA independent of the effect of the host-gene function. We found that the deletion of *mU50HG-b*-encoded mU50 snoRNA sequences resulted in a marked reduction in the quantity of mU50 snoRNA and methylation of the target rRNA, and that the perturbation of mU50 snoRNA was not crucial for growth and lifespan but yielded subtle anomalies and gene modulation in the lymphoid organs.

## Results

### Generation of ΔmU50_(HG-b)_ Mice and their Phenotype

**Figure 1:**
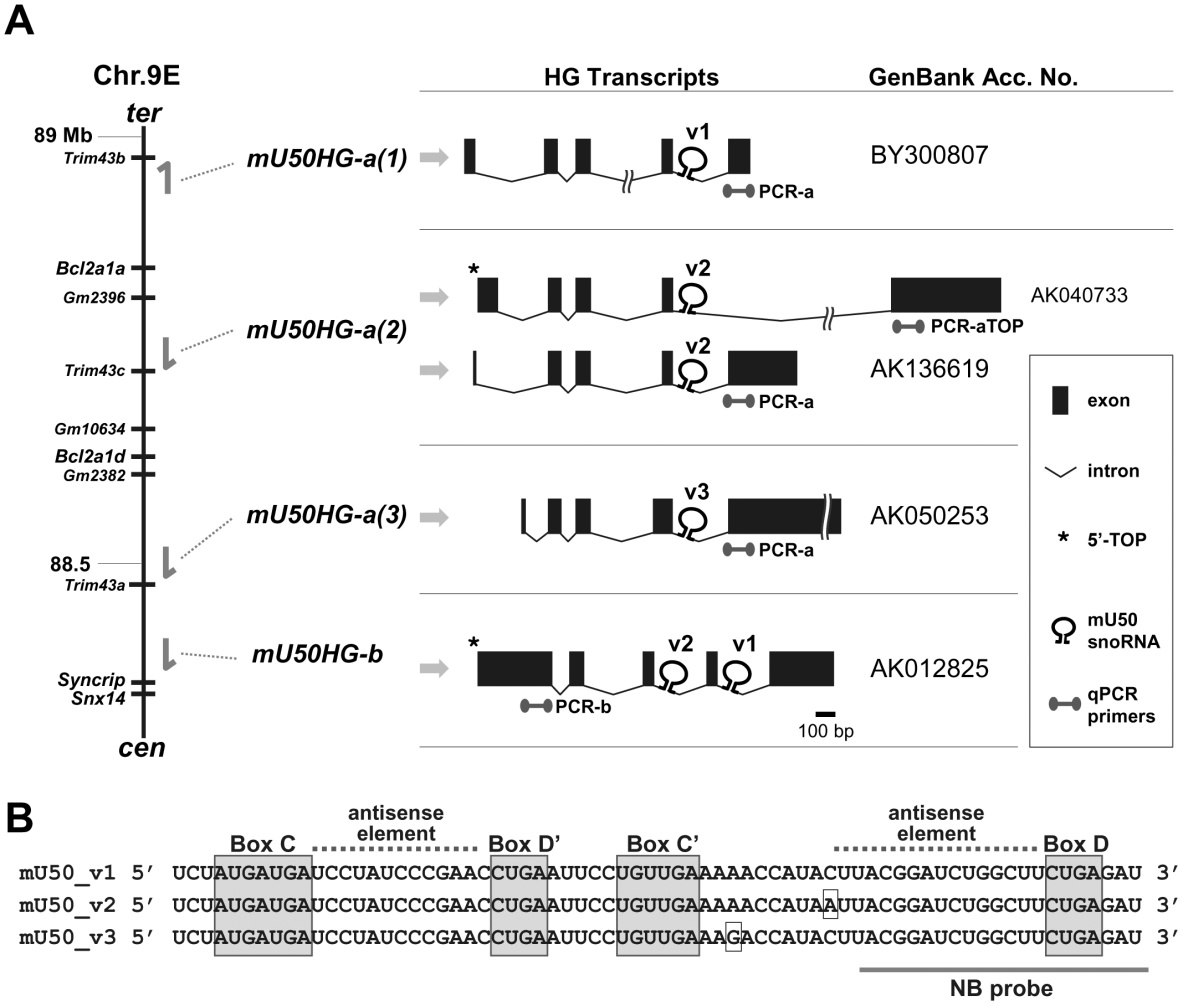
Genomic structure of mU50 host-genes on mouse chromosome 9. (A) Structure of transcripts for the mouse mU50 host-genes. Three *mU50HG-a*, *mU50HG-a(1)*, *-a(2)* and *-a(3)*, and the *mU50HG-b* loci are indicated sequentially by the arrows from the terminus (ter) to the centromere (cen) on the mouse chromosome. The mU50 host-genes except for *mU50HG-a(1)* are encoded in antisense direction. The right column illustrates the corresponding host-gene transcripts and their intron-encoded mU50 snoRNA variants, v1, v2, and v3. *mU50HG-b* contains mU50_v1 and v2 in distinct introns. Note that *mU50HG-a(2)* provides alternative splice variants. One transcript (AK040733) has a 5′TOP sequence (shown by asterisk) at the first exon, regarding our previous finding of the 5′TOP *mU50HG-a* gene [20]. The other splice variant (AK136619) possess the same sequence region at the fifth exon as the *mU50HG-a(1)* and *-a(3)*. We do not show an additional splice variant of *mU50HG-a(2)* (AK007093) listed in the GenBank database because the nucleotide length of the mU50-containing intron (17 kb) exceeds the appropriate length required for efficient processing of C/D-type snoRNA (≈85 nucleotides [45]). The primer sets used in qPCR analyses (Fig. 4) are also indicated. (B) Sequence of the mU50 variants, v1, v2 and v3. Conserved box sequences (C, D′, C′, and D) are indicated by gray rectangles. Two antisense elements, where the mU50 snoRNA interacts with the target rRNA, are indicated by broken lines. The single nucleotide polymorphisms among the variants are also shown by rectangles. NB: Northern blot.

To generate a U50 snoRNA deficient mouse model, we reinvestigated the genomic loci of mU50 host-genes in the C57BL/6 mouse genome in the NCBI database (http://www.ncbi.nlm.nih.gov/genome/). We found a *mU50HG-b* gene and a cluster of three distinct *mU50HG-a* gene structures (*mU50HG-a(1)*, *-a(2)*, and *-a(3)*) annotated on chromosome 9 (Fig. 1A). Sequence alignment of the host-genes and the corresponding transcripts revealed that *mU50HG-a(2)* was the 5′TOP *mU50HG-a* gene that we had reported previously [20]. In addition, when each of the intron-encoded mU50 snoRNA sequences in the *mU50HG-a* host-genes were compared, we found three single nucleotide polymorphisms in the middle of the snoRNA sequences that did not correspond to the complementary sequence to the rRNA target. We designated these mU50 snoRNA variants mU50_v1, mU50_v2, and mU50_v3 according to the host-gene name (Fig. 1B). *mU50HG-b* harbored two of these variants, mU50_v1 and mU50_v2, within the introns. Computer-assisted prediction of the RNA structures (MFold; http://mfold.rna.albany.edu/) revealed that these mU50 snoRNA sequence variants formed identical secondary structures in their most stable energy state (Fig. S1A). The sequences of the two antisense elements in mU50 snoRNA that interact with the target rRNA were conserved in the three mU50 snoRNA variants as well as in human U50 snoRNA (Fig. S1B).

**Figure 2:**
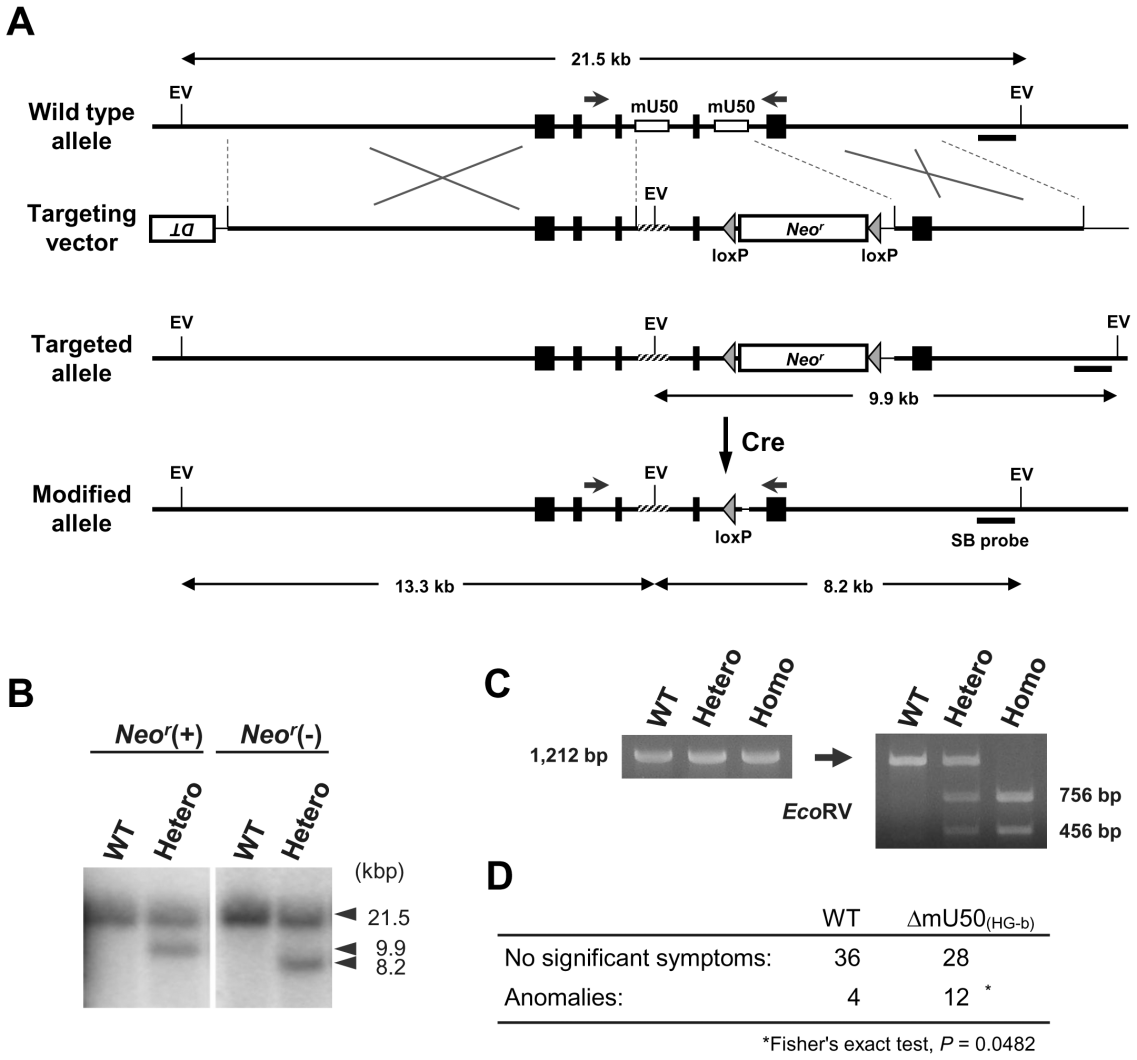
Generation of the mU50-deficient mice. (A) Schematic representation of the targeting strategy based on the wild-type allele. In the targeting vector, the first mU50 snoRNA sequence (upstream) is substituted by the 68 bp multiple cloning site of a pBluescriptII KSII plasmid containing an *Eco*RV recognition site (EV). The second mU50 snoRNA sequence (downstream) is replaced with a loxP-*Neo^r^*-loxP cassette. The pair of facing arrows indicates the set of forward/reverse primers used for genotyping PCR. *DT*: diphtheria toxin negative selection cassette; *Neo^r^*: neomycin resistant gene cassette; Cre: treatment with Cre recombinase; SB probe: a probe for Southern blot. (B) Southern blot of genomic DNA from mutant heterozygotes before (left) and after (right) removal of the *Neo^r^* cassette by Cre recombinase. WT: wild-type; Hetero: mutant heterozygotes. (C) Genotyping PCR for the *mU50HG-b* gene. Note that all the PCR products had nucleotide lengths that were identical to that of the wild-type. After digestion of the PCR product with *Eco*RV, fragmentation of the mutant allele-derived amplicon was observed. WT: wild-type; Hetero: mutant heterozygotes; Homo: ΔmU50_(HG-b)_ mutant. (D) Lifelong monitoring of the health condition (n = 40 per genotype) of ΔmU50_(HG-b)_ and wild-type mice. Note the greater number of anomalies in the ΔmU50_(HG-b)_ population. Detailed list is available in Table S1.

Gene targeting successfully generated heterozygous mice that possessed a mutant allele in which two mU50 snoRNA sequences located within the introns of the *mU50HG-b* gene were completely replaced by external sequences (Fig. 2A). The neomycin-resistant gene (1.7 kbp) was removed out by cross-breeding the heterozygous mice with CAG-Cre mice [21] in which Cre recombinase, which catalyzes recombination between two loxP sites, was expressed (Fig. 2B). The overall length and exon-intron structure of the reconstructed *mU50HG-b* gene were identical to those of the wild-type *mU50HG-b* (Fig. S2). By breeding the heterozygotes, we obtained homozygous mice that had a pair of the mutant alleles inherited maternally and paternally. A newly generated recognition site for the *Eco*RV restriction enzyme in the reconstructed *mU50HG-b* allowed the genotypes to be distinguished by PCR amplification followed by digestion of the PCR product with *Eco*RV (Fig. 2C). The mutant mice were born at Mendelian ratios and the ratio of female over male of 0.49 was comparable to the ratio of 0.48 found in the wild-type mice. We designated the mutant homozygotes as ΔmU50_(HG-b)_ mice. In the homozygous mating (n = 30), the average number of littermates was 5.9, similar to the 6.0 for the wild-type mice. The ΔmU50_(HG-b)_ mice appeared to grow normally, and were able to become pregnant, give birth, and feed their offspring.

Phenotypic characteristics of ΔmU50_(HG-b)_ mice, namely weight gain, wet weights of various organs, and peripheral blood chemistry and cytology were similar to those of wild-type (Figs. S3A–S3C). No significant difference in the cell proliferation activity was observed when the splenocytes isolated from the individual genotypes were grown in an *in vitro* culture system (Fig. S3D). In our lifelong monitoring of the health condition of the mice, we encountered a greater number of splenomegaly (an enlargement of the spleen) and swollen lymph nodes in the population of ΔmU50_(HG-b)_ mice when age-matched populations of both genotypes (from 35 to 98 weeks-old; n = 40 per genotype; Fisher’s exact test, *P* = 0.0482) were compared (Fig. 2D). However, no difference in tumor development was observed in both genotypes (Table S1).

### Expression of mU50 snoRNAs and mU50 Host-genes in ΔmU50_(HG-b)_ Mice

**Figure 3:**
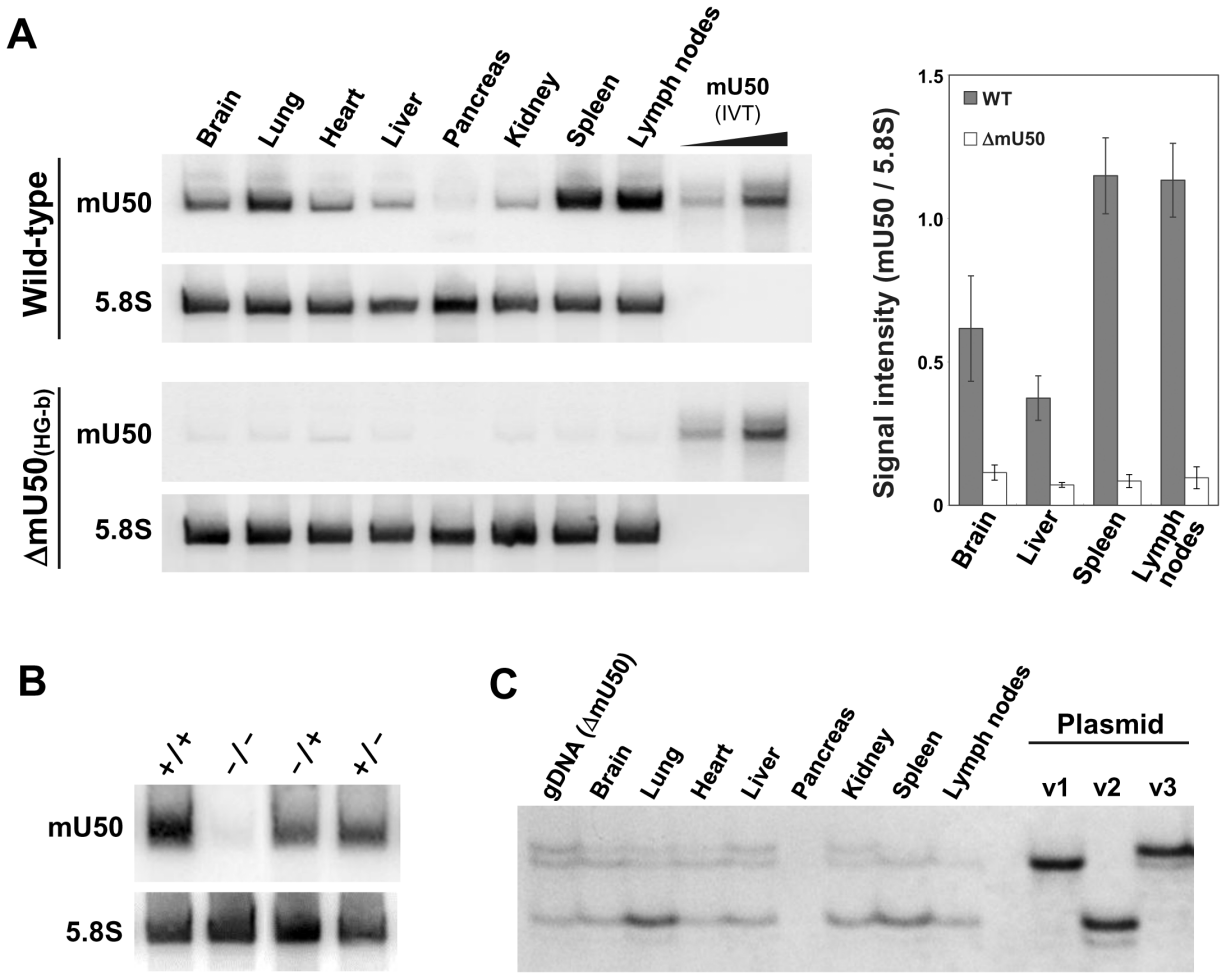
Molecular aspects of different organs in ΔmU50_(HG-b)_ and wild-type mice. (A) Northern blot analysis of mU50 snoRNA expression in eight organs from wild-type and ΔmU50_(HG-b)_ mice. *In vitro* transcribed (IVT) mU50 snoRNA was applied as a control. Detection of 5.8S rRNA was performed for the loading control. The signal intensities of selected organs are indicated in the panel on the right of the Figure. (B) Northern blot analysis of mU50 snoRNA and 5.8S rRNA expression in spleen obtained from wild-type (+/+), ΔmU50_(HG-b)_ (−/−), and maternally (−/+) and paternally (+/−)-inherited ΔmU50_(HG-b)_ heterozygotes. (C) PCR-SSCP analysis of the mU50 snoRNA variants in ΔmU50_(HG-b)_ mice. Genomic DNA from ΔmU50_(HG-b)_ mouse, which contains a single copy of each *mU50HG-a* paralog, was used as the control PCR template. gDNA: genomic DNA; v1, v2 and v3: plasmids that correspond to the mU50 snoRNAs encoded by *mU50HG-a(1)*, *-a(2)*, and *-a(3)*, respectively.

Northern blot analysis showed that, when compared with the age-matched wild-type mice, the intensity of the mU50 snoRNA signal was reduced markedly in all organs (i.e., brain, lung, heart, liver, pancreas, kidney, spleen, and lymph nodes) analyzed in 10-week-old ΔmU50_(HG-b)_ mice (Fig. 3A). The oligonucleotide probe that was used targets a region of mU50 snoRNA that is completely conserved in all three mU50 snoRNA variants as well as in human U50 snoRNA (Fig. 1B). It is notable that the mU50 snoRNA signals in wild-type mice were most prominent in the lymphoid organs (spleen and lymph nodes) (Fig. 3A, right). A comparison of the mU50 snoRNA signals in the spleen of wild-type, ΔmU50_(HG-b)_-homozygous and heterozygous mice showed that the mU50 snoRNA signal was reduced by half in heterozygous mice, but no difference was found between mice with either the maternally (−/+) or paternally (+/−) inherited mutant allele (Fig. 3B). We also confirmed that the mU50 snoRNA-deficient condition was maintained in the corresponding organs in 60-week-old ΔmU50_(HG-b)_ mice (data not shown).

As illustrated in Fig. 1A, the mU50 host-genes, *mU50HG-a(1)*, *-a(2)*, and *-a(3)*, encode each of the discrete mU50 snoRNA variants, namely, mU50_v1, mU50_v2, and mU50_v3, respectively, while the *mU50HG-b* host-gene encodes two mU50 sequences that correspond to the mU50_v1 and mU50_v2 snoRNAs. We found that the abundant expression of the *mU50HG-b*-derived mU50_v1 and v2 snoRNAs was eliminated in ΔmU50_(HG-b)_ mice and, therefore, sought to clarify the distribution of the mU50 snoRNA variants that were expressed from the *mU50HG-a* genes among the various organs in the ΔmU50_(HG-b)_ mice using PCR-SSCP (single-strand conformation polymorphism). Analysis of the genomic DNA extracted from ΔmU50_(HG-b)_ mouse embryos supported the presence of single copies of each of the *mU50HG-a* gene variants (Fig. 3C, the first lane). In addition, we found that the proportion of the mU50 snoRNA variant-derived signals varied in individual organs (Fig. 3C): mU50_v2 was predominant in lung and spleen; both mU50_v1 and mU50_v2 were predominant in brain and heart; and all three variants were evenly expressed in liver and kidney. This finding indicates that the *mU50HG-a*-derived mU50 snoRNA variants also showed organ-specific expression patterns, although the organ specificities for the *mU50HG-a*-derived variants were different from those elucidated for the *mU50HG-b*-derived snoRNAs.

**Figure 4:**
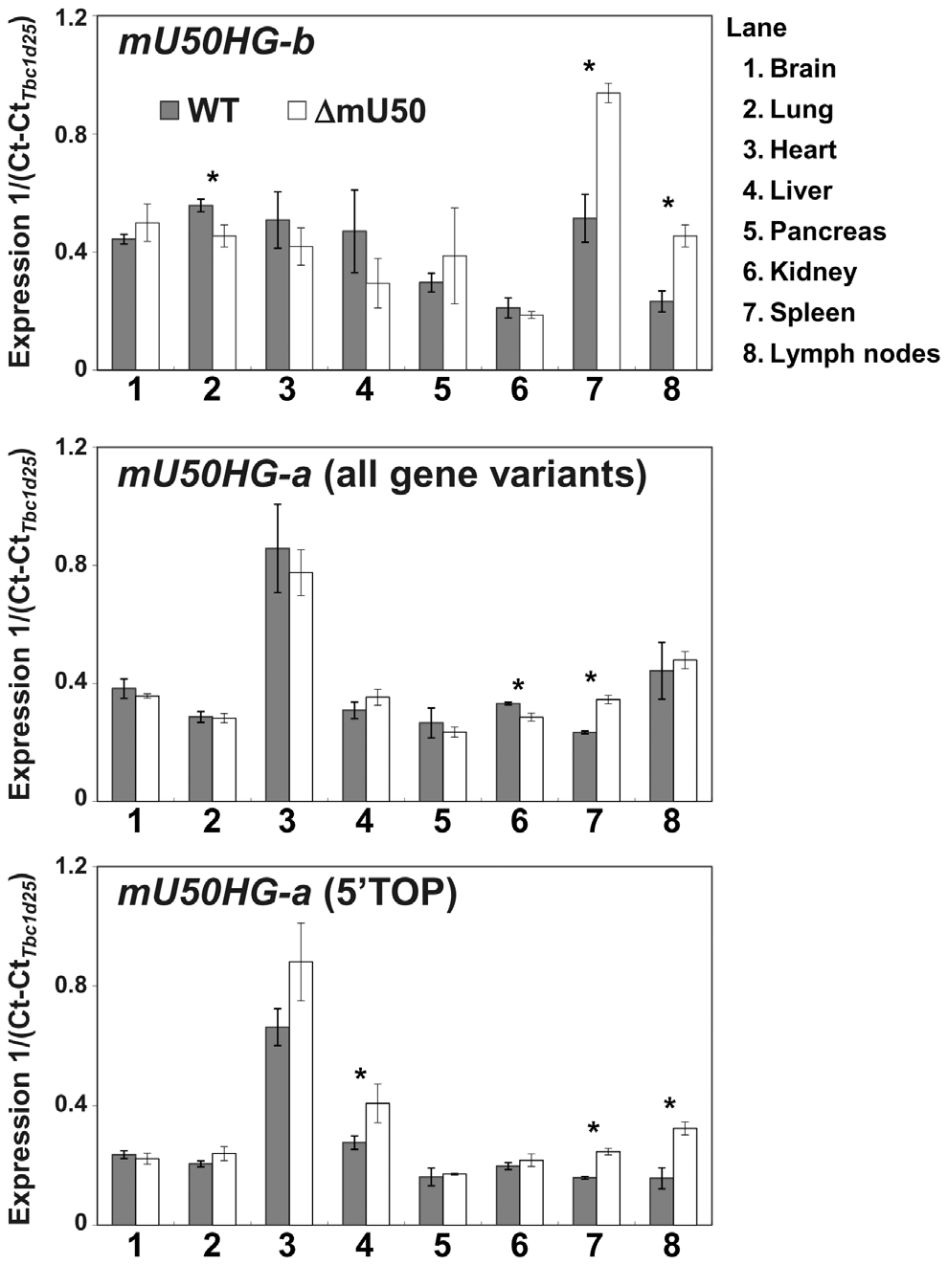
Expression profiles of mU50 host-genes in eight organs from wild-type and ΔmU50_(HG-b)_ mice. Real-time qPCR for determination of the discrete mU50 host-genes were conducted using three primer sets: PCR-b primer set for detection of *mU50HG-b* transcript, PCR-a primer set for amplification of all transcripts from the three *mU50HG-a* loci, and PCR-aTOP primer set for *mU50HG-a(2)* with 5′TOP sequence (see Fig.1A). The threshold value was normalized using the reference gene (*Tbc1d25*). See Materials and methods on the selection of reference genes for mouse organs. Error bars = 1 S.D. for three biological replicates. *P<0.05; WT: wild-type; ΔmU50: ΔmU50_(HG-b)_ mice.

To analyze the expression levels of the mU50 host-genes by quantitative PCR (qPCR), we designed three sets of primers to differentially detect *mU50HG-b*, *mU50HG-a* (all three gene variants), and a 5′TOP transcript of *mU50HG-a(2)* (Fig. 1A). In wild-type mice, the substantial expression of *mU50HG-b* was observed in the all organs analyzed; the expression levels were higher in brain, lung, heart, liver, and spleen, and lower in pancreas, kidney, and lymph nodes (Fig. 4, top). Regarding the expression levels of the *mU50HG-a* variants, the use of two primer sets yielded comparable results: the highest level in heart and lower levels in all the other organs analyzed (Fig. 4, middle and bottom). In ΔmU50_(HG-b)_ mice, it is notable that *mU50HG-b* expression increased significantly in the lymphoid organs, whereas the corresponding host-gene expression in the other organs was comparable to their expression in the wild-type mice (Fig. 4, top). The expression profiles of *mU50HG-a* host-genes in ΔmU50_(HG-b)_ mice were similar to those in wild-type mice (middle), but the expression of the 5′TOP transcript of *mU50HG-a(2)* increased significantly in both spleen and lymph node of ΔmU50_(HG-b)_ mice compared with in wild-type, as was observed for the 5′TOP *mU50HG-b* transcript (Fig. 4, bottom).

### Influence of *mU50HG-b*-derived mU50 snoRNA Depletion on Cellular Function in ΔmU50_(HG-b)_ Mice

**Figure 5:**
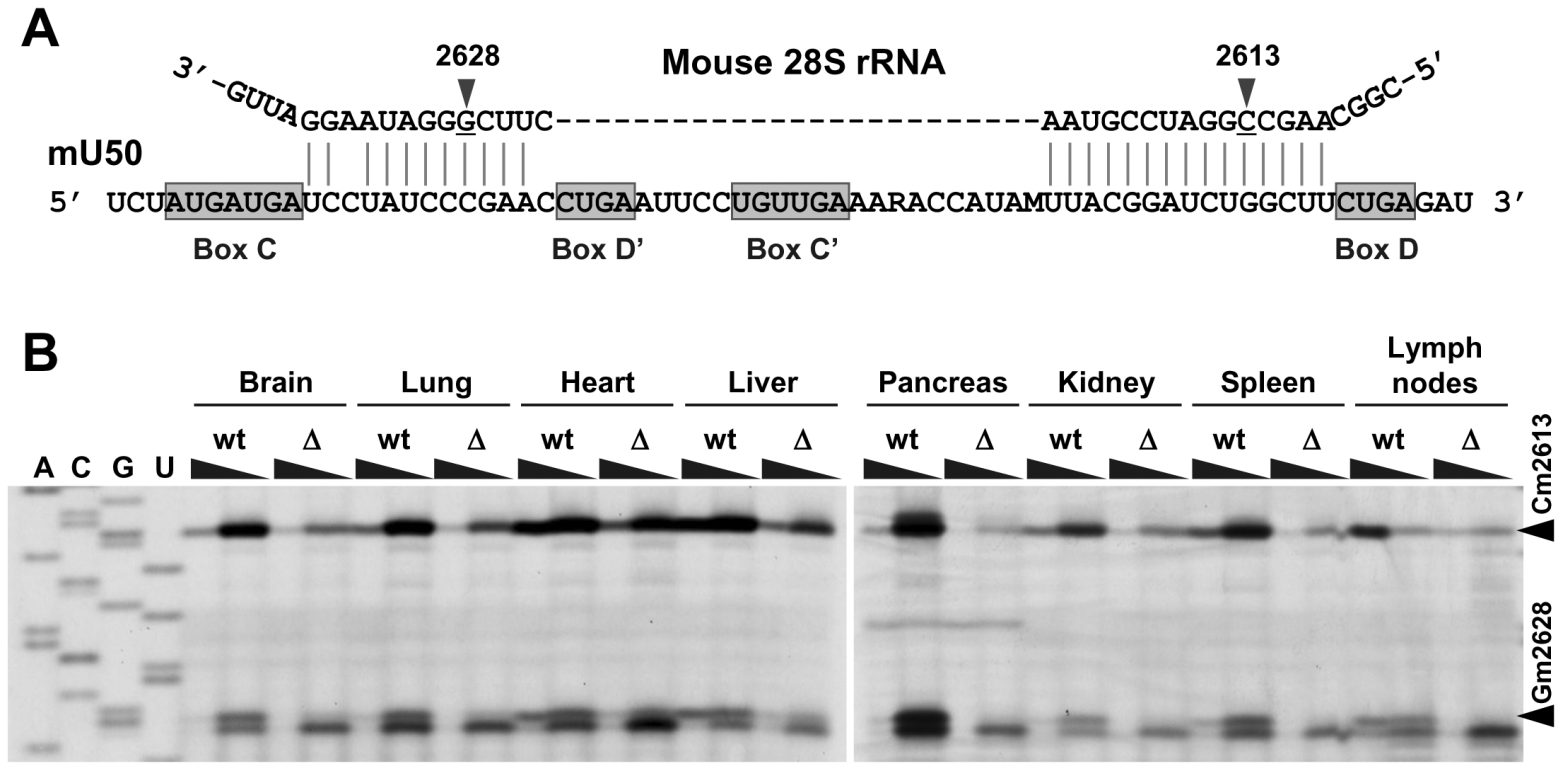
Mechanism of action of mU50 snoRNA. (A) Schematic representation of mU50 snoRNA binding to 28S rRNA. The mU50 snoRNA sequence contains two sites that are complementary to two methylation-target sites in the 28S rRNA sequence. Consensus C and D (and C’ and D’) box motifs are shown in shaded rectangles. The ribonucleotides that are located 5 bases prior to boxes D and D’ (indicated by the arrows) are methylated. (B) Methylation-sensitive primer extension assay for U50-sites in various mouse organs. The wedges across every two lanes indicate the concentration of dNTP (1 mM and 0.004 mM) in the reaction mix. The reverse transcriptase generates a stop signal one nucleotide before the 2′-O-methylated nucleotide in the presence of 0.004 mM dNTP. The upper and lower arrows on the right of the Figure indicate the Cm2613 and Gm2628 stop signals on 28S rRNA, respectively. wt: wild-type; Δ: ΔmU50_(HG-b)_ mice.

To evaluate whether the reduced amount of mU50 snoRNA affected methylation status of the target rRNA in the cells, a primer extension assay was performed for mU50 snoRNA-target sites on 28S rRNA (Fig. 5A). In this assay, a deoxynucleotide concentration as low as 0.004 mM made the reverse transcriptase methylation-sensitive and this resulted in the cessation of polymerization one nucleotide before a methylated nucleotide. We found that the resulting stop signals on the 28S rRNA (Cm2613 and Gm2628) were largely eliminated in all the studied organs of the ΔmU50_(HG-b)_ mice, showing that the box C/D mU50 snoRNAs caused the loss of canonical rRNA modification (Fig. 5B).

**Figure 6:**
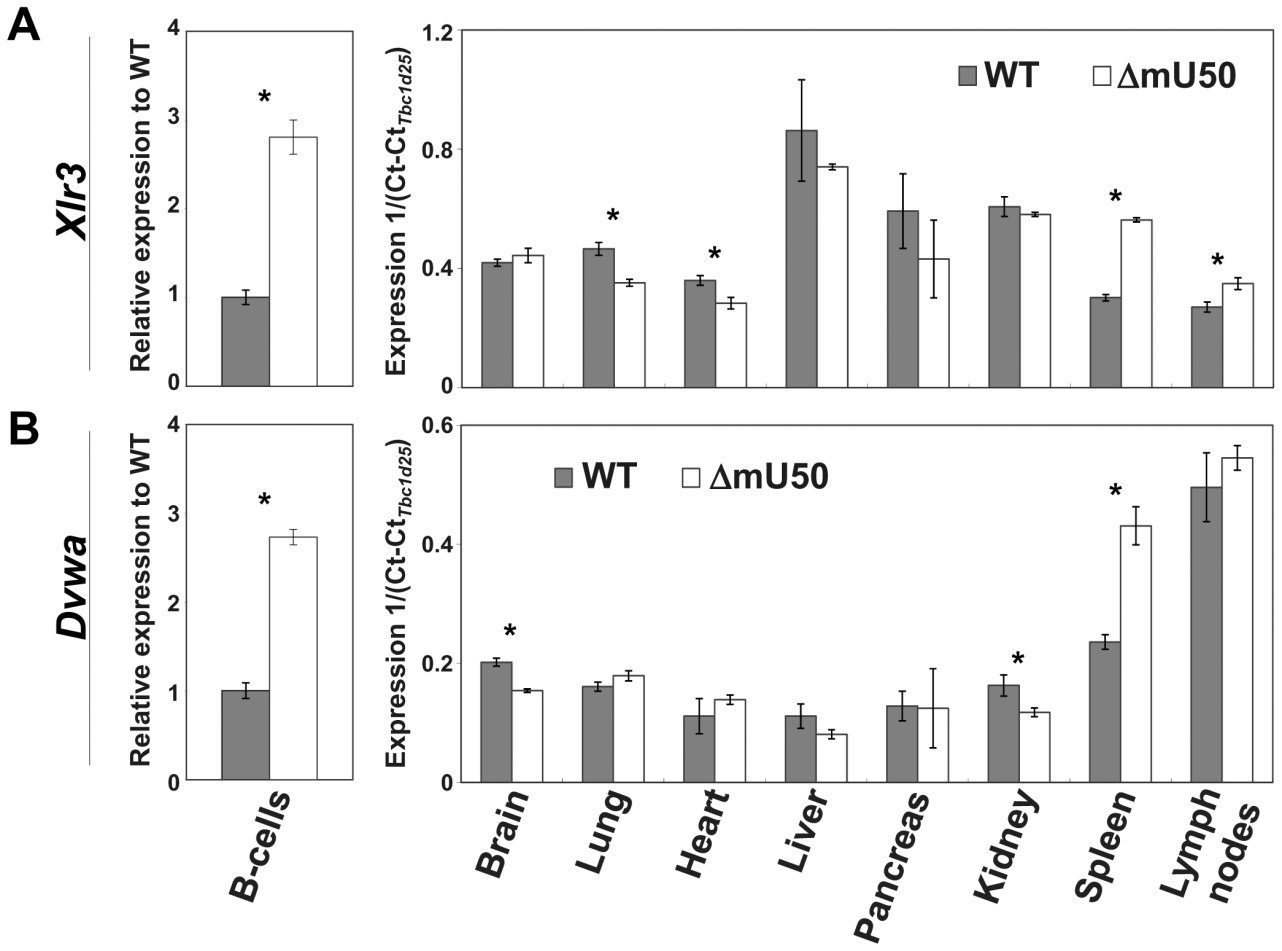
Expression analysis of *Xlr3a* and *Dvwa* genes. TaqMan®-based real-time qPCR was conducted to validate *Xlr3a* (A) and *Dvwa* (B) expression in splenic B-lymphocytes and eight organs. The threshold value was normalized by the reference gene (*Tbc1d25*). Error bars = 1 S.D. for three biological replicates. *P<0.05; WT: wild-type; ΔmU50: ΔmU50_(HG-b)_ mice.

To assess the influence of reduced mU50 snoRNA status on the overall gene expression profile, we conducted a microarray-based analysis using isolated splenic B-lymphocytes (Table 1). We found 42 genes that were differentially expressed (>1.5-fold) between the wild-type and ΔmU50_(HG-b)_ mice, including immunoglobulin (Ig) genes and several heat shock protein (Hsp) family genes. The qPCR analysis validated the significant up-regulation of X-linked lymphocyte-regulated 3A (*Xlr3a*) and dual von Willebrand factor A (*Dvwa*; also known as collagen VI alpha 4 gene *Col6a4*) in ΔmU50_(HG-b)_ splenic B-lymphocytes (Fig. 6). Notably, *Xlr3a* was up-regulated in spleen and lymph nodes in ΔmU50_(HG-b)_ mice, while it remained stable or was down-regulated in the other organs analyzed (Fig. 6A). *Dvwa* was also up-regulated in spleen, but not in lymph nodes, in ΔmU50_(HG-b)_ mice (Fig. 6B). In a preliminary 2D electrophoresis in combination with peptide mass fingerprinting analysis, the only differentially expressed proteins that were detected were peptides derived from Hsp70 and an actin-like unnamed protein product (data not shown). Gene expression analysis also showed that *c-Myc*, a possible transcriptional regulator of human *U50HG* gene [22], and *Bcl6*, a transcriptional repressor in relation to translocation in human lymphoma [17], were detected at similar expression levels in the wild-type and ΔmU50_(HG-b)_ mice (Fig. S4).

**Table 1:** Differentially expressed genes between wild-type and ΔmU50_(HG-b)_ mouse B-lymphocytes detected by a comparative microarray analysis.

Gene Symbol	Gene Title	Ratio	Z-score
**Up-regulated in ΔmU50_(HG-b)_ mice**			
*Dvwa* *	dual von Willebrand factor A domains	2.56	10.64
*Xlr3a*	X-linked lymphocyte-regulated 3A	2.63	9.27
*Slc15a2*	solute carrier family 15 (H+/peptide transporter), member 2	2.24	9.17
*Hspa1b*	heat shock protein 1B	1.86	7.06
*Hsph1*	heat shock 105 kDa/110 kDa protein 1	1.77	6.54
*Cpt1a*	carnitine palmitoyltransferase 1a, liver	1.69	5.98
*Siglech*	sialic acid binding Ig-like lectin H	1.85	5.91
*Mgl1*	macrophage galactose N-acetyl-galactosamine specific lectin 1	1.77	5.51
*Ddx6*	DEAD (Asp-Glu-Ala-Asp) box polypeptide 6	1.53	4.87
*Klk1*	kallikrein 1	1.65	4.86
*Ugcg*	UDP-glucose ceramide glucosyltransferase	1.50	4.68
*Hspa4l*	heat shock protein a4-like	1.60	4.56
*Cd209d*	CD209d antigen	1.59	4.46
*Strn3*	striatin, calmodulin binding protein 3 (probed in intron)	1.57	4.37
*A230046K03Rik*	RIKEN cDNA A230046K03 gene	1.55	4.23
*Lifr*	leukemia inhibitory factor receptor	1.55	4.21
*Slc5a3*	solute carrier family 5 (inositol transporters), member 3	1.54	4.18
*Hspa1a*	heat shock protein 1A	1.70	4.11
*2810442I21Rik*	RIKEN cDNA 2810442I21 gene	1.69	4.07
*Tbc1d8*	TBC1 domain family, member 8	1.52	4.06
*Rbm25*	RNA binding motif protein 25	1.55	3.40
**Down-regulated** **in ΔmU50_(HG-b)_ mice**			
*Gm13051*	Zinc finger-containing	0.49	−6.68
*Fscn1*	fascin homolog 1, actin bundling protein	0.49	−5.73
*Sdc1*	syndecan 1	0.59	−4.89
*Chst1*	carbohydrate (keratan sulfate Gal-6) sulfotransferase 1	0.60	−4.81
*Ly6k*	lymphocyte antigen 6 complex, locus K	0.62	−4.52
*Idi1*	isopentenyl-diphosphate delta isomerase	0.62	−4.52
*Lman1*	lectin, mannose-binding, 1	0.62	−4.47

* also known as *Col6a4* (collagen, type VI, alpha 4).

Genes that exhibit >1.5-fold up/down-regulation between wild-type and ΔmU50_(HG-b)_ mice are listed. Probes target to immunoglobulin genes are omitted from the list (see Materials and Methods; complete dataset is available on online GEO database under the accession number GSE41164).

## Discussion

We have successfully generated a mouse line that shows significant reduction of mU50 snoRNA expression without modification of the host-gene structure (Fig. 2 and 3A). In previous studies, snoRNA-knockout mouse models were established in relation to the etiology of a human neurodegenerative disorder, Prader-Willi syndrome [23], [24]. In these mouse models, more than 40 copies of intron-encoded box C/D-type snoRNA, SNORD116 (formerly HBII-85), and exons of the non-coding host-gene were removed by targeted deletion of a huge genomic locus (≈150 kb). In this respect, the ΔmU50_(HG-b)_ mouse generated in this study is the first model in which a single methylation-guide snoRNA is eliminated without altering the genomic structure of the host-gene.

### Reduction of mU50 snoRNA Caused by Gene-deletion was not Causative for Tumorigenicity but was Responsible for Alterations in the Lymphoid Organs

The lifelong monitoring of age-matched genotypes showed no marked alterations in phenotypic characteristics of ΔmU50_(HG-b)_ mice, such as weight gain, wet weights of various organs, and cell proliferation activity in an *in vitro* culture system (Fig. S3). There was no genotype-related difference in the incidence of tumor development in the whole body but, in connection with the abundant mU50 snoRNA expression in the lymphoid organs in wild-type mice, splenomegaly and swollen lymph nodes were observed more frequently in ΔmU50_(HG-b)_ mice (Fig. 2D and Table S1).

Our microarray and qPCR analysis revealed altered expression levels of some genes, for example, *Xlr3a* and *Dvwa*, in ΔmU50_(HG-b)_ B-lymphocytes (Table 1 and Fig. 6). The expression of the *XLR* (X-linked lymphocyte-regulated) gene family is highly correlated with characteristics of a mature phenotype in B-lymphocytes [25]. Mouse *Dvwa* was recently cloned as an equivalent to a collagen type VI gene *Col6a4*, and RT-PCR analysis in adult mice showed the *Dvwa* was highly expressed in ovary, intestine, and spleen [26]. Our qPCR analysis showed that both *Xlr3a* and *Dvwa* were up-regulated markedly in ΔmU50_(HG-b)_ spleen, whereas their expression levels in the other ΔmU50_(HG-b)_ organs analyzed were either unaffected or down-regulated (Fig. 6). Regarding the putative interaction between mU50 snoRNA and gene transcripts, it is notable that both *Xlr3a* and *Dvwa* mRNAs contain multiple sequences (≈12 nucleotides) that are complementary to the mU50 snoRNA (data not shown). Other studies have suggested multiple roles of snoRNAs in post-transcriptional events such as alternative splicing, A-to-I RNA editing, and RNA chaperone activity, in addition to their modification-guidance role [27]–[29]). Whether mU50 snoRNAs contribute to the post-transcriptional regulation of these genes still needs to be explored; however, our findings coupled with the abundant mU50 snoRNAs support the possible functional importance of mU50 snoRNA in lymphoid organ physiology.

Gene expression profiling showed that genes encoding Hsp were up-regulated in ΔmU50_(HG-b)_ mice (Table 1). The Hsp family is known to contain molecular chaperones that are expressed under various cellular stresses and they interact with misfolded proteins causing them to re-folding correctly [30]. In yeast, ribosomes deficient in rRNA modifications succumb to altered translational fidelity supporting the proposed importance of snoRNA-mediated rRNA modifications in optimizing rRNA structure for the production of accurate and efficient ribosomes [31]. In our ΔmU50_(HG-b)_ animal model, we showed that the depletion of mU50 snoRNA resulted in the reduced methylation status of the corresponding rRNA (Fig. 5). By extrapolating from the tertiary structure of *Thermus thermophilus* ribosomes [32], we found that the two target sites of U50 snoRNA on the rRNA (U50-sites; identical in both human and mouse) were located at the inter-subunit bridge of 23S rRNA (corresponding to 28S rRNA in vertebrates) (Fig. S1C). The inter-subunit bridge is responsible for the attachment between the large and small ribosomal subunits and is distant from the peptidyl transferase center that is a core region for the ribozyme activity of ribosomes [33]. We postulate that the U50-sites are not critical for protein synthesis but that they might disturb the harmonic motion between ribosomal subunits, leading to perturbations in protein synthesis. From a phylogenetic point of view, it is of interest that the corresponding U50-sites in rRNAs are highly conserved through bacteria to vertebrates (Fig. S1C), but no methylation occurs at the corresponding sites in bacteria and yeast because of a lack of orthologous U50 snoRNA in these non-vertebrate classes [34]. Taken together, we hypothesize that U50 snoRNA-mediated methylation might be relatively newly required in vertebrates, and it may be particular important in fine-tuning regulation of the hematopoietic system and lymphoid function concomitant with the development of acquired immunity. In this context, we speculate that the ribosomes in lymphoid organs need a higher amount of mU50 snoRNA for the abundant production of immunoglobulin, and therefore, upon substantial reduction in mU50 snoRNA in our ΔmU50_(HG-b)_ model, the proteins that are translated by the modified ribosomes might require a chaperone machinery that is supported by the induced Hsp proteins.

### Differential Expression Patterns of mU50 Host-genes and mU50 snoRNA among Different Organs

The results of mU50 host-gene expression obtained by qPCR revealed that together the mU50 host-genes represent a substantial amount of the transcripts in all the organs analyzed in both wild-type and ΔmU50_(HG-b)_ mice (Fig. 4). Of most interest was the organ-dependency of *mU50HG-b* expression, for example, higher in heart and liver and lower in lymph nodes, which is inconsistent with the organ-specific distribution of mU50 snoRNA, for example, their enrichment in spleen and lymph nodes and their sparseness in heart and liver (Fig. 3A). In general, the processing of non-protein-coding host-gene transcripts are identified and rapidly degraded in cells by nonsense-mediated decay (NMD) [35], although it has also been documented that snoRNA host-gene transcripts display variable susceptibility to NMD [36]. Thus, a possible scenario is that the mU50 host-gene transcripts are particularly susceptible to NMD in some organs, as has been shown for the *U87HG* transcript [36]; alternatively, the mU50 snoRNAs in non-lymphoid organs might be eliminated rapidly by RNA degradation machinery. Regarding RNA degradation, recent studies using deep sequencing provided evidence that snoRNA products are processed by RNA degradation machinery, which generates smaller miRNA-like molecules in various types of cells [37]–[39]. At present, little is known about the degradation machinery for mU50 snoRNA and host-genes in various organs; clearly, further studies are required to elucidate this.

The differential expression profiles of mU50 host-genes imply that mU50 host-genes *per se* may play a role in some organs. Recent studies documented that some non-protein-coding snoRNA host-genes themselves can be active players in cell-cycle regulation. For example, *Gas5* was shown to be involved in the regulation of cell death and proliferation in breast cancer, and its reduced expression was associated with poor prognosis in the studied patients [40]. Cell cycle regulation with the *Gas5* transcript has also been demonstrated in human T-lymphocytes [41]. Another non-protein-coding snoRNA host-gene, *ZFAS1*, which is highly expressed in the human mammary gland, was found to be down-regulated in breast cancer [42]. When the mouse ortholog, *Zfas1*, was down-regulated by siRNA in a mouse mammary cell line, cell proliferation and differentiation were activated without affecting the expression of the snoRNA hosted within its intron [42]. The results obtained from our cell culture experiment indicated that the proliferation activity of ΔmU50_(HG-b)_ mice-derived splenocytes was comparable to that of the wild-type splenocytes (Fig. S3D). At this point, we cannot rule out the possibility that mU50 host-genes might have a potential role in cell cycle progression.

### U50 snoRNA-releasing Systems in Mouse and Human

Mice possess four mU50 snoRNA-supplying host-genes, three *mU50HG-a* genes and one *mU50HG-b* in their genome, while human have only a single copy of *U50HG* [17]. We previously annotated the *mU50HG-b* gene as a *U50HG* ortholog because of the structural similarity between the two genes. The present reinvestigation of the promoter sequences (from −2,000 to +500 bp flanking the transcription start site) of mU50 host-genes and human *U50HG* further supported the close relationship between *mU50HG-b* and *U50HG* (Fig. S6). In relation to the multiplicity of mU50 host-genes, we initially speculated that *mU50HG-a*-derived mU50 snoRNA might be sufficient to compensate for the depletion of *mU50HG-b*-derived mU50 snoRNA in ΔmU50_(HG-b)_ mice. Northern blot analysis, however, indicated that this is not the case; the amount of mU50 snoRNA in ΔmU50_(HG-b)_ mice, which should have been derived from the *mU50HG-a* genes, remained very low and did not show a lymphoid organ-specific pattern (Fig. 3A). We also took advantage of the absence of *mU50HG-b*-derived mU50 snoRNAs in ΔmU50_(HG-b)_ mice to explore the expression status of *mU50HG-a*-derived mU50 snoRNAs by PCR-SSCP. As a result, we found that the three mU50 snoRNA variants from the three individual *mU50HG-a* genes were differentially expressed among the studied organs but none of them were specific to the lymphoid organs (Fig. 3C). Taking these findings together, we propose that the expression of *mU50HG-b* in mouse, and *U50HG* in human, might contribute uniquely to the enrichment of mU50 snoRNAs in the wild-type lymphoid organs, and the *mU50HG-a* genes in mouse might be regulated independently from *mU50HG-b* expression and may simply have an additional effect on the total amount of mU50 snoRNAs in the corresponding organs. It should be noted that *mU50HG-b* expression was up-regulated in the lymphoid organs of ΔmU50_(HG-b)_ mice when mU50 snoRNA expression was substantially reduced (Fig. 4). The observation that the 5′TOP *mU50HG-a* transcript showed similar responses implied that the 5′TOP motif might provide an additional cue for mU50 host-genes to be expressed in the lymphoid organs. Details of the molecular mechanisms that may be involved remain to be explored.

In conclusion, we generated ΔmU50_(HG-b)_ mice that lack *mU50HG-b*-derived mU50 snoRNAs in the whole body giving us a unique model of a single snoRNA knockdown with intact genomic host-gene structure and expression. Despite the significant reduction of mU50 snoRNAs and the 2′-*O*-ribose methylation of the target rRNA, the ΔmU50_(HG-b)_ mice exhibited normal growth and lifespan. Our major finding is that the influence of mU50 snoRNA depletion, albeit small or mild, appeared mostly in the lymphoid organs where the mU50 snoRNAs are most abundant in the wild-type animals. A comparison of the expression patterns of mU50 snoRNA and host-genes suggests degradation-resistance of mU50 snoRNAs in lymphoid organs. Hence, in the future, it is important to elucidate the discrete transcriptional regulation of the multiple mU50 host-genes as well as the processing and maintenance of the mU50 snoRNAs for a more detailed understanding of their physiological roles in animals.

## Materials and Methods

All the procedures in the present study that involved animals were reviewed and approved by the Research Center for Odontology Section of Biological Sciences in the Nippon Dental University, Japan (Permit Number: 10–18). Statistical analysis was performed using the StatView software version 5.0 (SAS Institute Inc., USA).

### Targeting Vector Construction and Generation of an mU50-deficient Mouse Line

We performed long-accurate PCRs for the sequences up- and down-stream of the *mU50HG-b* gene using a clone from the RPCI-22 BAC library segment 1 (Roswell Park Cancer Institute, USA). The PCR products were used as homologous arms in the targeting vector. The diphtheria toxin (DT) cassette, loxP sites, and the neomycin resistance gene (*Neo^r^*) were subcloned from a pBl-lx-Neo-DT vector. E14.1 derived from 129P2/Ola (obtained from Dr. Nobuaki Yoshida, Institute of Medical Science, The University of Tokyo) were electroporated with 25 µg of a linearized mU50 targeting vector per 10^7^ cells. Embryonic stem (ES) cells were then plated onto mitomycin C-treated G418-resistant primary mouse embryonic fibroblast cells and selected with G418. Resistant colonies were picked up 6–8 days after selection. Homologous recombination was screened for by PCR and confirmed by Southern blot analysis. Chimeric mice were produced by an aggregation method as described previously [43]. Ten to 15 ES cells were aggregated with C57BL/6J x DBF1 eight-cell stage embryos in a hole on a plastic dish and cultured overnight. Then the well-formed blastocysts were transferred into the uterus of pseudopregnant female mice. Next, the male chimera mice were bred with C57BL/6J female mice, and germline transmission was checked by agouti coat color. Mice heterozygous for the mU50 mutation were crossed with C57BL/6-Tg(CAG-cre) mice to remove the *Neo^r^* cassette flanked by loxP sites from the mU50 targeted locus [21]. The mice obtained were then backcrossed to the C57BL/6J genetic background for at least six generations. We used sixth generation mutant mice for most of the experiments and 10th generation for microarray and gene expression analyses.

### Animals and Cells

Heterozygous mice were interbred or bred to C57BL/6J mice. All breeding was performed in a conditioned room (25°C, 60% humidity, and a 12∶12 hour light-dark cycle) at the animal facility of Nippon Dental University, Tokyo, Japan.

A dissected spleen was gently ground by two slide-glasses and collected into a test tube with Hanks’ balanced salt solution (HBSS; Invitrogen, USA). The cell pellet of the splenocytes was treated with 4 ml of ACK lysing buffer (Lonza, Germany) for 5 min to lyse out the erythrocytes. After several washings of the cells with HBSS, FITC-conjugated anti-CD45R/B220 antibody (BD Biosciences, USA) was added and reacted for 15 min on ice. Non-reacted antibody was washed out with HBSS, and then the cells were reacted with anti-FITC antibody-coupled magnetic beads (Miltenyi Biotec, USA) for 10 min. After rinsing twice, B-cells were separated using a LS+MACS separation column (Myltenyi Biotec).

### Northern Blot Analysis

Total RNA was purified from mouse organs or isolated cells using ISOGEN reagent (Nippongene, Japan) according to the manufacturer’s instructions. In brief, 10–20 µg of total RNA were denatured for 3 min at 95°C in 1×RNA buffer (49% formamide, 0.02% SDS, and 0.015% bromophenol blue) and separated on a denaturing gel (10% polyacrylamide (3.3% cross-linker), 7 M urea, and 1×TBE). Electrophoresis was performed at 12 W. Transfer onto a Hybond-N+ membrane (GE Biosciences, USA) was performed by electroblotting using a semidry blotting apparatus (TransblotSD, Bio-Rad, USA) at 2 mA/cm^2^ for 35 min in 0.5×TBE. RNA was immobilized onto the membrane by UV cross-linking with 120 mJ in a UV Stratalinker (Stratagene, USA). An oligonucleotide probe was 5′-labeled with [γ-^32^P]-ATP using T4 polynucleotide kinase (Promega, USA). Hybridization was carried out at 42°C in hybridization buffer (1 M sodium phosphate (pH 6.2) and 7% (w/v) SDS) for 16 h. Blotted membranes were washed twice for 10 min at 25°C in washing solution I (2×SSC, 0.1% (w/v) SDS) followed by a second washing step for 10 min with washing solution II (0.1×SSC, 0.1% (w/v) SDS). Membranes were exposed to a storage phosphor screen (Amersham) for 10–16 h and were scanned using a Typhoon image analyzer system (GE Biosciences). An oligonucleotide probe (5′-ATCTCAGAAGCCAGATCCGT-3′), which is complementary to the 3′-side of mU50 snoRNA sequence, was designed to hybridize all three mU50 snoRNA variants (Fig. 1B), and the oligonucleotide probe (5′-TCCTGCAATTCACATTAATTCTCGCAGCTAGC-3′), which is complementary to 5.8S rRNA, was used as the loading control. Densitometry was performed using ImageJ 1.45 (http://rsbweb.nih.gov/ij/).

### Primer Extension Assay

Ten micrograms of total RNA with 0.5 pmol oligodeoxynucleotide primer that was conjugated with either AlexaFluor®488 or fluorescein (FAM) was heat denatured for 1 min at 95°C. Following a 10 min hybridization step at 43°C, reverse transcription was carried out in a buffer containing 200 units of M-MLV reverse transcriptase (Promega, USA) in the presence of either 1 mM dNTP or 0.004 mM dNTP. For the sequencing reactions, dideoxynucleotides were added to a final concentration of 0.06 mM. cDNA products were ethanol precipitated and primer extension products were resolved on a denaturing gel (6% polyacrylamide and 8 M urea) and visualized by scanning using a Typhoon Analyzer (Amersham). For sequencing ladders, a control rRNA gene sequence was obtained by PCR amplification of C57BL/6J genomic DNA with specific primers for the U50-sites (from 2537 bp to 2662 bp of the 28S rRNA gene), and the PCR product was cloned into a TA vector (Promega, USA). The primers that were used are listed in Table S2.

### Real-time qPCR

RNA samples were treated with DNase I (Invitrogen), and then converted to cDNA using an oligo-dT primer and the SuperScript® First-strand synthesis system for RT-PCR (Invitrogen). qPCR was carried out using TaqMan® gene expression assay (ABI Inc., USA) and SYBR Green I® PCR Master Mix (ABI) with a Prism 7000 Real-time PCR System (ABI). Three technical replicates were run per sample. For the SYBR-based qPCR analysis, we tried several sets of PCR primers for each gene. The efficiency of each primer set was tested with serial dilutions of cDNA, and the combination of primers that yielded the best single amplified product was selected by checking for a single sharp peak in the dissociation curve of each product. The cycle threshold value (Ct) of each target gene was normalized relative to the reference genes (see below). All primer sets, except those for the TaqMan® assays, were designed using Primer3 via the NCBI webpage (http://www.ncbi.nlm.nih.gov/tools/primer-blast/) and are listed in Table S2.

To select appropriate reference genes for the survey of gene expression in various organs, we used the RefGenes tool from the Genevestigator platform [44] and selected the three top-ranking genes, *Tbc1d25*, *Bud13*, and *Krt81* (Fig. S5A). Microarray analysis confirmed that these genes were expressed equally in wild-type and ΔmU50_(HG-b)_ mice and that their signal intensities were at adequate levels but not as high as the signal intensity of the house-keeping *Actb* gene (Fig. S5B). We failed to obtain optimal amplification conditions for *Krt81*, but qPCR analyses showed that both *Tbc1d25* and *Bud13* yielded comparable expression levels (a reciprocal number of Ct value) among the eight organs analyzed (Fig. S5C). Based on these findings, we used *Tbc1d25* as a reference in all the qPCR analyses in this study.

### PCR-SSCP

The cDNA library was constructed using a miScript system (QIAGEN, Germany) according to the manufacturer’s instruction. Control plasmids containing individual mU50 variants were cloned into a pGEM-T easy vector (Promega). PCR primers for the mU50 sequence (forward, 5′-TCTATGATGATCCTATCCCG-3′; reverse, 5′-ATCTCAGAAGCCAGATCCGT-3′) were labeled with either AlexaFluor®647 or 6-carboxyfluorescein (6-FAM) at the 5′-ends. In the PCR reaction, 2 ng of template cDNA was used and amplified for 30 cycles. The PCR product was diluted to 1∶125 with a loading buffer (96% deionized formamide containing 10 mM EDTA, 0.01% bromophenol blue), denatured at 95°C for 10 min, and applied to an acrylamide sequencing gel (10% native PAGE (aa:bis = 19∶1) in 1×TBE). The gels were run at 1000 V (25V/cm) for 190 min at 22°C and scanned using a Typhoon scanner (GE Biosciences).

### DNA Microarray

Microarray analysis was performed at the core facility of Cell Innovator Inc. (Fukuoka, Japan). Total RNA from splenic B-cells (23 weeks old; three samples per genotype) was purified with RNeasy Micro kit (QIAGEN). The quality of the RNA was assessed by monitoring the absorbance at 260 and 280 nm and rRNA fragmentation was measured using a Bioanalyzer (Agilent Technologies, USA). Concentrations of purified RNA samples were determined using a Nanodrop spectrophotometer (ND-1000; Thermo Scientific, USA) and equal amounts of the three RNA samples were mixed. cRNA was hybridized to a GeneChip® Microarray (Mouse Expression 430 2.0 Array, Affymetrix, USA) containing 45,101 probes that cover more than 20,000 mouse genes. The expression value and detection calls were computed from the raw data following the procedures outlined for the Affymetrix Microarray Suite software version 5.0. A complete gene list of the microarray analyses was built using GeneSpring software version 7.3.1 (Silicon Genetics Inc., USA). Because arranged immunoglobulin (Ig) genes could not be distinguished in the mixed samples, we omitted the probes for Ig genes from the analysis.

### Data Deposition

The microarray data have been deposited and are available online in the Gene Expression Omnibus (GEO) database (http://www.ncbi.nlm.nih.gov/geo/) under the accession number GSE41164.

## Supporting Information

Figure S1 **Structural bases of mU50 snoRNA.** (A) Computer-assisted prediction of the secondary structure of mU50 snoRNA variants. The k-turn structure that might possibly be formed by box C and D motifs was not taken into account for the prediction. Note that the all three variants exhibit the identical structure at the most stable energy states. (B) Sequence similarity among human and mouse U50 snoRNAs. Human U50A and U50B snoRNAs (formerly U50 and U50’ in [17]) are compared with the mU50 snoRNA sequence. Conserved box motifs are indicated by rectangles. Antisense elements to 28S rRNA are indicated by broken lines. The four U residues in the U50A snoRNA (shown in red and underscored) are where a genomic TT-deletion has been reported in prostate and breast cancers [18], [19]. (C) Schematic representation and nucleotide sequences of the mU50 snoRNA target sites (in orange) on the rRNA gene in five organisms. The segments down-stream of the mU50-sites (gray rectangles) are variable having expanded in size through insertions and/or duplications during the evolutionary process. A further comparative genomic data analysis indicated that the U50-sites are highly conserved from archaea to vertebrate. Referring to a previous report of the *T. thermophilus* 23S rRNA structure [32], the conserved site for the mU50-sites is subject to form the loop 62 which participates in the formation of the inter-subunit bridge.
(PDF)

Figure S2 **Nucleotide sequences of the modified** ***mU50HG-b*** **gene in comparison with the original sequence in wild-type.** The sequence starts from the intron 3 which contains an mU50 snoRNA sequence (upstream) through the intron 4 where another mU50 sequence (downstream) resides. mU50 snoRNA and exon sequences are shown in blue and black background, respectively. Nucleotides unaltered upon the recombination are connected with asterisks. The upstream mU50 sequence is completely replaced with a non-coding sequence derived from pBluescript II plasmid, and the downstream mU50 sequence is replaced with residual nucleotides of the loxP-*Neo* ^r^-loxP cassette after the Cre-lox recombination. Note that the sizes of the modified introns are identical to those of wild-type.
(PDF)

Figure S3 **Phenotypes of the mU50-deficient animals.** (A) Weight gain of female and male ΔmU50_(HG-b)_ and wild-type mice (n = 14 per genotype). A plot of ΔmU50_(HG-b)_ mice was always lower than that of the wild-type mice throughout the observation period, although this difference was not statistically significant. Error bars = 1. S.D. (standard deviation) (B) Average tissue weight (% body-weight) in wild-type and ΔmU50_(HG-b)_ mutant mice at 10 weeks after birth (n = 7 per genotype). Although some organs such as heart, liver, and testis of ΔmU50_(HG-b)_ mice were lighter than the same organs in wild-type, no prominent differences were found in their morphology and histology (data not shown). Error bars = 1 S.D. *P<0.05. (C) Age-associated changes in the number of peripheral lymphocytes (n = 4 per genotype) in ΔmU50_(HG-b)_ and wild-type mice. Fitted curves for ΔmU50_(HG-b)_ (broken line) and wild-type (solid line) are indicated. (D) Proliferation activity of splenocytes *in vitro*. For cell culture, 2×10^5^ of isolated splenocytes from individual genotypes were grown on 6-well plates (BD Falcon, USA) in the presence of 1.0 μg/ml of lipopolysaccharides (Sigma, USA) as a stimulus. The numbers of cells were counted with a hemocytometer. The number of cells 24 h after inoculation was designated as 100%. Error bars = 1 S.D. from three independent assays.
(PDF)

Figure S4 **Representative data of qPCR validation of relative gene expression in splenocytes between wild-type and ΔmU50_(HG-b)_ mice.** The threshold value was normalized by the beta-actin gene (*Actb*) according to the ΔΔCt method. Error bars = 1 S.D. for three biological replicates. *P<0.05; WT: wild-type; Δ: ΔmU50_(HG-b)_ mice.
(PDF)

Figure S5 **Selection of qPCR reference genes for assessment of organ-dependent gene expression patterns.** (A) A result view of RefGenes platform [44] on inquiry for appropriate reference genes for qPCR survey to eight organs. The 3 top-ranking candidates were *Tbc1d25*, *Bud13*, and *Krt81*. (B) Comparable expression levels of the candidate genes obtained from microarray analysis of splenic B-cells in wild-type and ΔmU50_(HG-b)_ mice. (C) TaqMan®-based qPCR analyses of the candidate genes in various organs of wild-type mice. The expression levels (a reciprocal number of Ct value) of reference genes among eight organs are compared. Note that both *Tbc1d25* and *Bud13* showed less inter-organ variations in their expression levels as compared to those of *Actb.*
(PDF)

Figure S6 **Comparative genomic analyses of human and mouse mU50 host-gene promoter sites.** (A) The phylogenetic tree was constructed based on the promoter sequences (from −2,000 bp behind 5′TOP transcription start site to +500 bp) of these U50 snoRNA host-genes using VISTA tools for comparative genomics that is provided by Lawrence Berkeley National Laboratory (http://genome.lbl.gov/vista/index.shtml). The results support the closest promoter sequence of *mU50HG-b* to human *U50HG*. (B) Conservation of promoter sequences among human and mouse U50 host-genes. Highly conserved loci (>70% similarity) with human *U50HG* are shown in pink.
(PDF)

Table S1 **Anomalies detected in age-matched wild-type and ΔmU50_(HG-b)_ mice.** Age-matched mice that were euthanized or had died were examined pathologically (n = 40 per genotype).
(DOC)

Table S2 **List of PCR primers used in this study.**
(DOC)
