# Supplementary figures and images for: Generation of a Mouse Model with Down-Regulated U50 snoRNA (SNORD50) Expression and Its Organ-Specific Phenotypic Modulation
Source: PLoS One. 2013 Aug 26;8(8):e72105. doi: 10.1371/journal.pone.0072105 (PMC3753356; doi:10.1371/journal.pone.0072105)

A

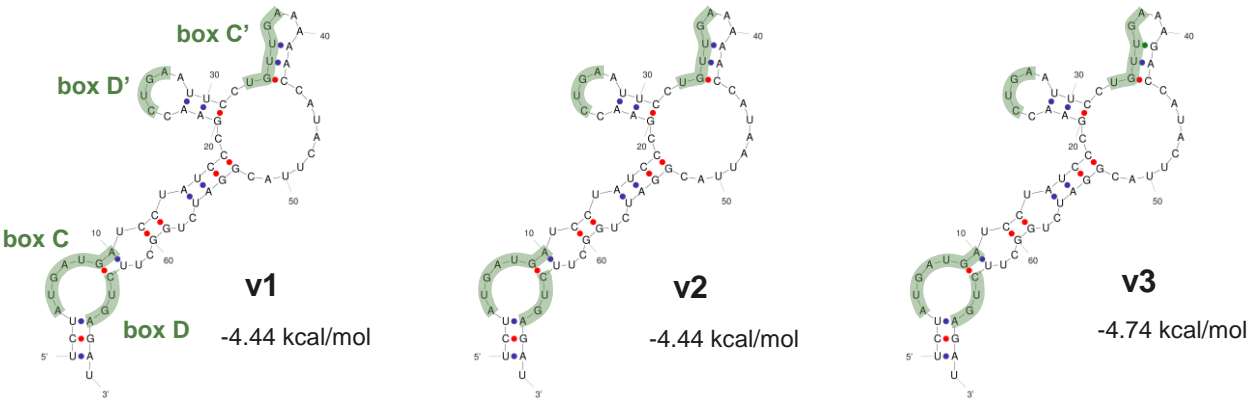

B

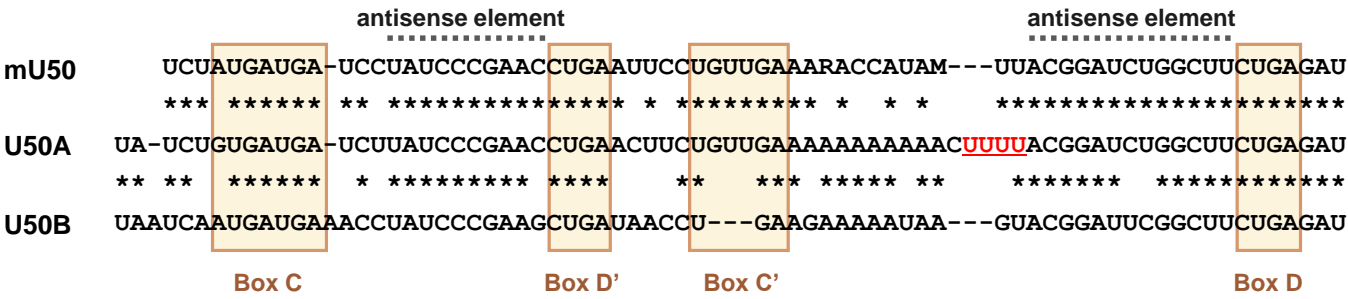

C

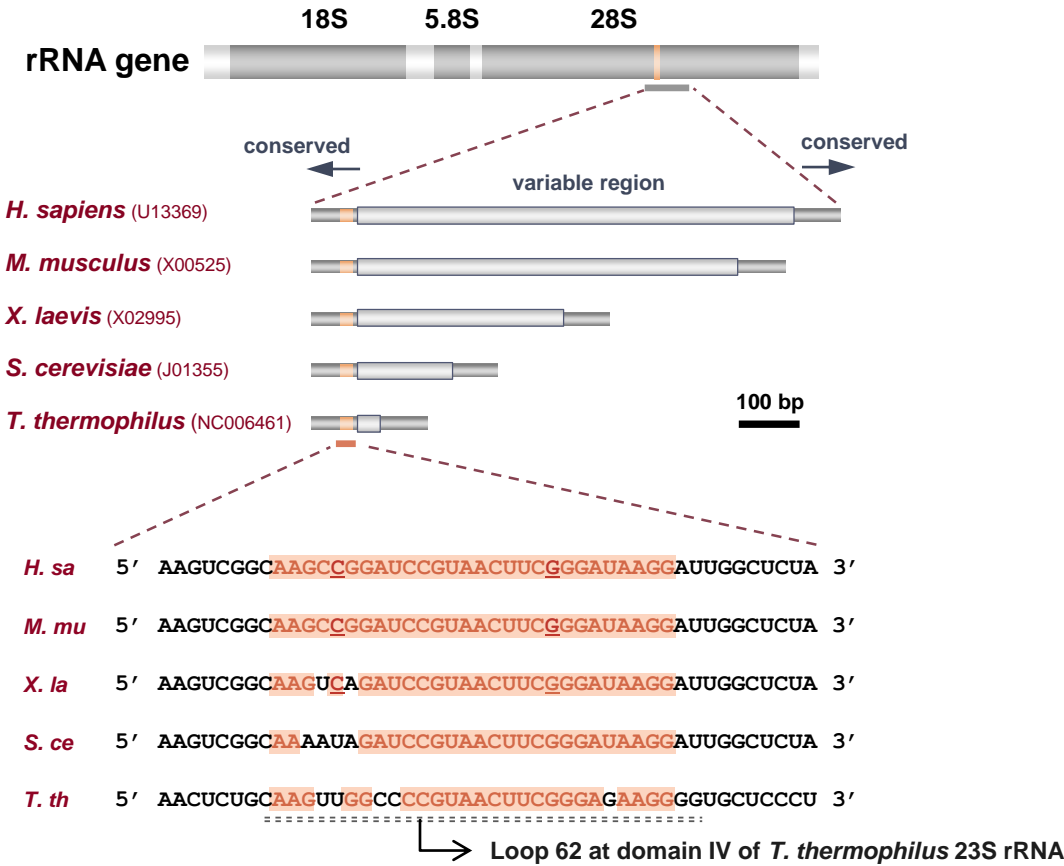

Supplement: Figure S1 — Structural bases of mU50 snoRNA. (A) Computer-assisted prediction of the secondary structure of mU50 snoRNA variants. The k-turn structure that might possibly be formed by box C and D motifs was not taken into account for the prediction. Note that the all three variants exhibit the identical structure at the most stable energy states. (B) Sequence similarity among human and mouse U50 snoRNAs. Human U50A and U50B snoRNAs (formerly U50 and U50’ in [17]) are compared with the mU50 snoRNA sequence. Conserved box motifs are indicated by rectangles. Antisense elements to 28S rRNA are indicated by broken lines. The four U residues in the U50A snoRNA (shown in red and underscored) are where a genomic TT-deletion has been reported in prostate and breast cancers [18], [19]. (C) Schematic representation and nucleotide sequences of the mU50 snoRNA target sites (in orange) on the rRNA gene in five organisms. The segments down-stream of the mU50-sites (gray rectangles) are variable having expanded in size through insertions and/or duplications during the evolutionary process. A further comparative genomic data analysis indicated that the U50-sites are highly conserved from archaea to vertebrate. Referring to a previous report of the T. thermophilus 23S rRNA structure [32], the conserved site for the mU50-sites is subject to form the loop 62 which participates in the formation of the inter-subunit bridge. (PDF) [file pone.0072105.s001.pdf]

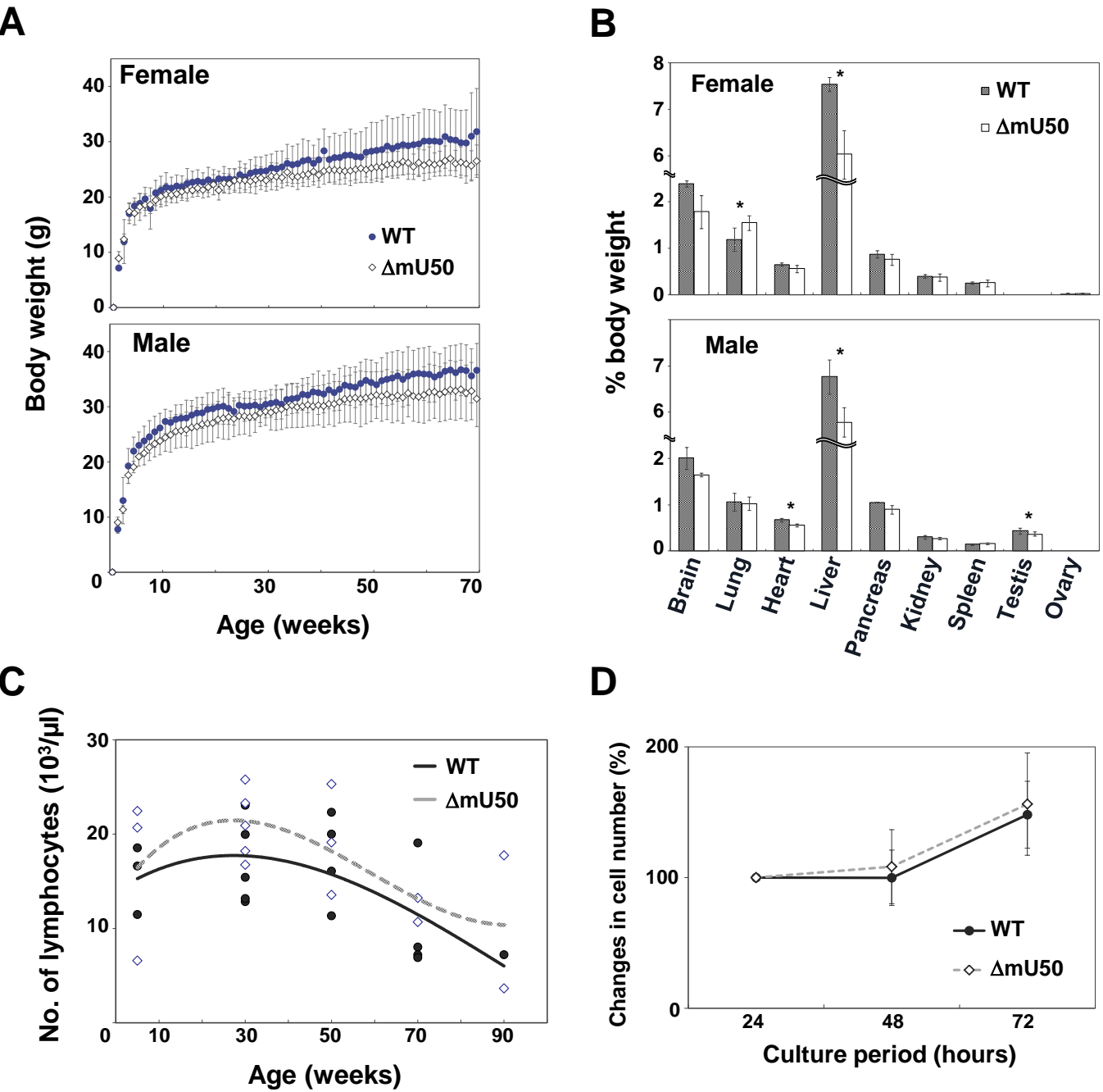

Supplement: Figure S3 — Phenotypes of the mU50-deficient animals. (A) Weight gain of female and male ΔmU50(HG-b) and wild-type mice (n = 14 per genotype). A plot of ΔmU50(HG-b) mice was always lower than that of the wild-type mice throughout the observation period, although this difference was not statistically significant. Error bars = 1. S.D. (standard deviation) (B) Average tissue weight (% body-weight) in wild-type and ΔmU50(HG-b) mutant mice at 10 weeks after birth (n = 7 per genotype). Although some organs such as heart, liver, and testis of ΔmU50(HG-b) mice were lighter than the same organs in wild-type, no prominent differences were found in their morphology and histology (data not shown). Error bars = 1 S.D. *P<0.05. (C) Age-associated changes in the number of peripheral lymphocytes (n = 4 per genotype) in ΔmU50(HG-b) and wild-type mice. Fitted curves for ΔmU50(HG-b) (broken line) and wild-type (solid line) are indicated. (D) Proliferation activity of splenocytes in vitro. For cell culture, 2×10^5^ of isolated splenocytes from individual genotypes were grown on 6-well plates (BD Falcon, USA) in the presence of 1.0 μg/ml of lipopolysaccharides (Sigma, USA) as a stimulus. The numbers of cells were counted with a hemocytometer. The number of cells 24 h after inoculation was designated as 100%. Error bars = 1 S.D. from three independent assays. (PDF) [file pone.0072105.s003.pdf]

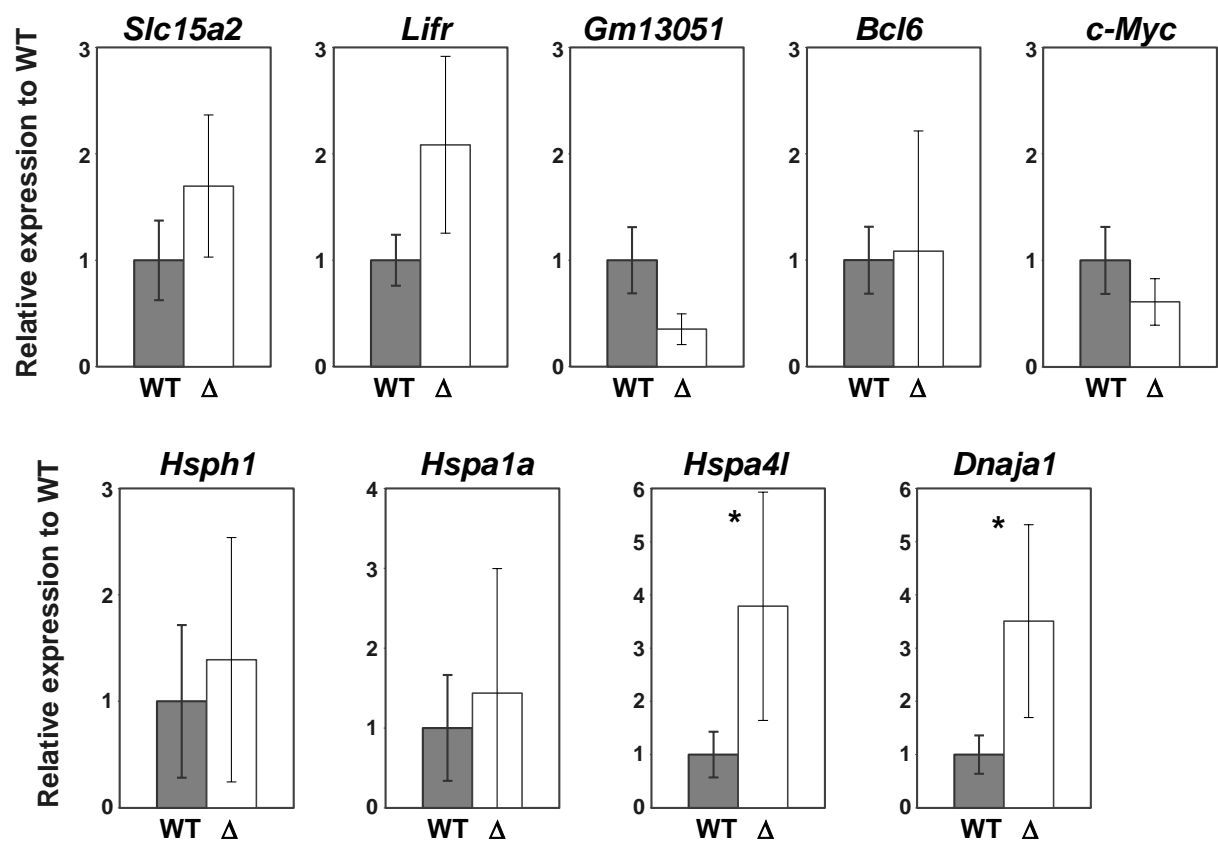

Supplement: Figure S4 — Representative data of qPCR validation of relative gene expression in splenocytes between wild-type and ΔmU50(HG-b) mice. The threshold value was normalized by the beta-actin gene (Actb) according to the ΔΔCt method. Error bars = 1 S.D. for three biological replicates. *P<0.05; WT: wild-type; Δ: ΔmU50(HG-b) mice. (PDF) [file pone.0072105.s004.pdf]

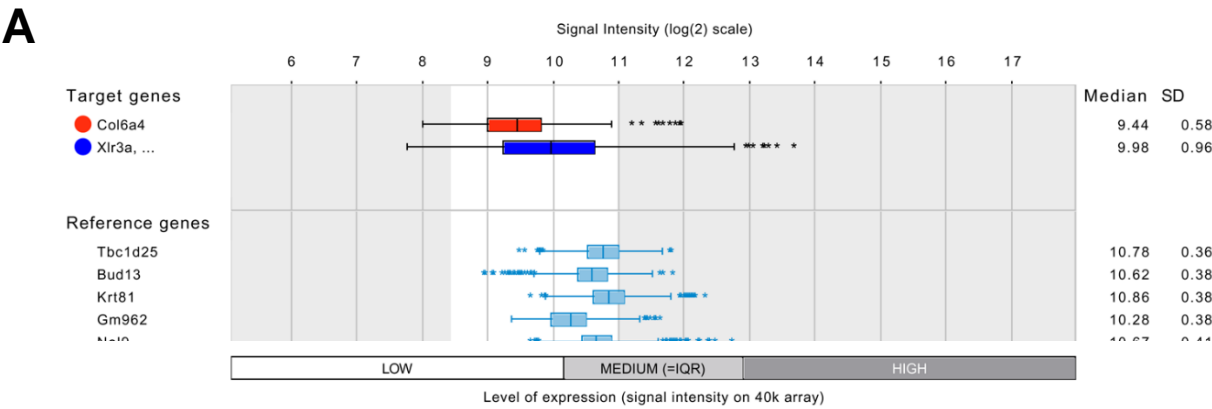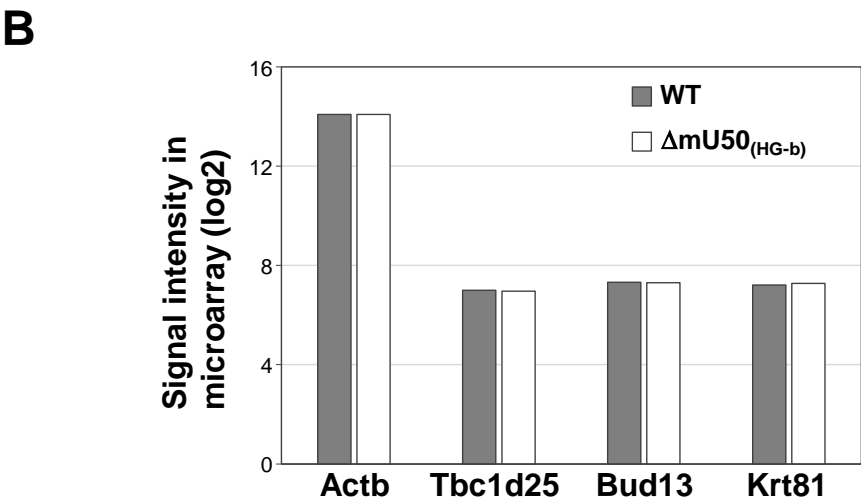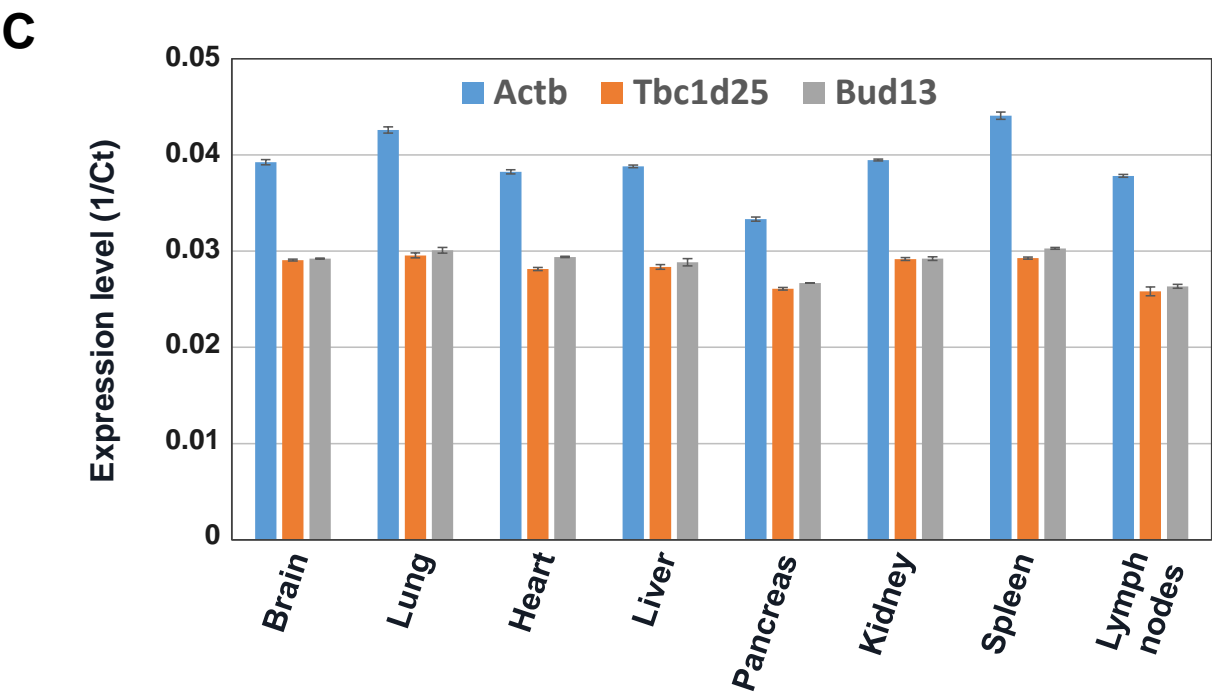

Supplement: Figure S5 — Selection of qPCR reference genes for assessment of organ-dependent gene expression patterns. (A) A result view of RefGenes platform [44] on inquiry for appropriate reference genes for qPCR survey to eight organs. The 3 top-ranking candidates were Tbc1d25, Bud13, and Krt81. (B) Comparable expression levels of the candidate genes obtained from microarray analysis of splenic B-cells in wild-type and ΔmU50(HG-b) mice. (C) TaqMan®-based qPCR analyses of the candidate genes in various organs of wild-type mice. The expression levels (a reciprocal number of Ct value) of reference genes among eight organs are compared. Note that both Tbc1d25 and Bud13 showed less inter-organ variations in their expression levels as compared to those of Actb. (PDF) [file pone.0072105.s005.pdf]

A

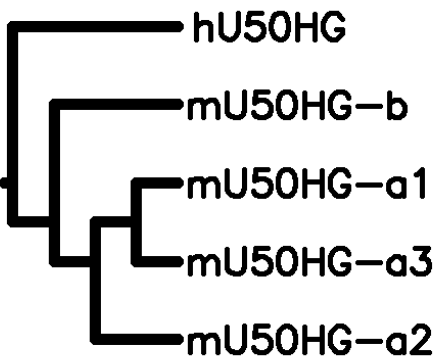

B

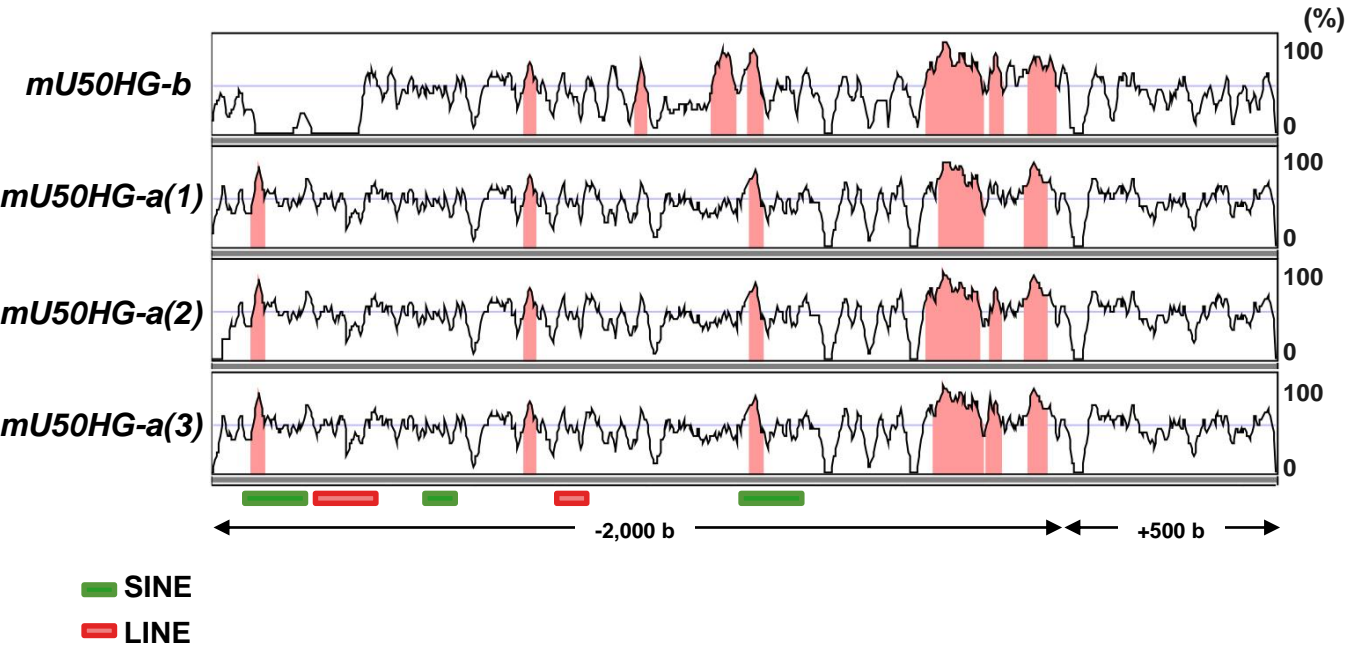

Supplement: Figure S6 — Comparative genomic analyses of human and mouse mU50 host-gene promoter sites. (A) The phylogenetic tree was constructed based on the promoter sequences (from −2,000 bp behind 5′TOP transcription start site to +500 bp) of these U50 snoRNA host-genes using VISTA tools for comparative genomics that is provided by Lawrence Berkeley National Laboratory (http://genome.lbl.gov/vista/index.shtml). The results support the closest promoter sequence of mU50HG-b to human U50HG. (B) Conservation of promoter sequences among human and mouse U50 host-genes. Highly conserved loci (>70% similarity) with human U50HG are shown in pink. (PDF) [file pone.0072105.s006.pdf]
