# Supplementary material for: Generation of a Mouse Model with Down-Regulated U50 snoRNA (SNORD50) Expression and Its Organ-Specific Phenotypic Modulation
Source: PLoS One. 2013 Aug 26;8(8):e72105. doi: 10.1371/journal.pone.0072105 (PMC3753356; doi:10.1371/journal.pone.0072105)
Supplement: Figure S2 — Nucleotide sequences of the modified mU50HG-b gene in comparison with the original sequence in wild-type. The sequence starts from the intron 3 which contains an mU50 snoRNA sequence (upstream) through the intron 4 where another mU50 sequence (downstream) resides. mU50 snoRNA and exon sequences are shown in blue and black background, respectively. Nucleotides unaltered upon the recombination are connected with asterisks. The upstream mU50 sequence is completely replaced with a non-coding sequence derived from pBluescript II plasmid, and the downstream mU50 sequence is replaced with residual nucleotides of the loxP-Neo r-loxP cassette after the Cre-lox recombination. Note that the sizes of the modified introns are identical to those of wild-type. (PDF) [file pone.0072105.s002.pdf]

Supplementary Figure S2

Soeno et al.

```
        Intron 3                                              mU50
WT      gtgagtggtgctctgttttgttttctgctgagcttactgtcgttggtctagTCTATGATGATCCTATCCC
        ***************************************************
ΔmU50   gtgagtggtgctctgttttgttttctgctgagcttactgtcgttggtctagatcgataagcttgatatcg
                                                                     EcoRV

        GAACCTGAATTCCTGTTGAAAAACCATAATTACGGATCTGGCTTCTGAGatggaccattgcaaaggacta
                                                         *********************
        aattcctgcagcccggggatccactagttctagagcgcgcgcatcgatatggaccattgcaaaggacta

                                                                     Exon 4
        ttgtgttcttctttaatgttggcaatgacgtaatgcctgtgtttttgtttttatattttagGGCCAGTGA
        **********************************************************************
        ttgtgttcttctttaatgttggcaatgacgtaatgcctgtgtttttgtttttatattttagGGCCAGTGA

                                                                  Intron 4
        CACAAGAACTTGCGAGAGATGCCAGTCCGCCTGTGAAGATCCTGCAGCAGGAGTGCATgtgagtggtgct
        **********************************************************************
        CACAAGAACTTGCGAGAGATGCCAGTCCGCCTGTGAAGATCCTGCAGCAGGAGTGCATgtgagtggtgct

                                               mU50
        ctgttttgttttctgctgagcttactgtcgttggtctagTCTATGATGATCCTATCCCGAACCTGAATTC
        ************************
        ctgttttgttttctgctgagcttaaagcttgatgggtaccgagcgaacgcgtataacttcgtatagcata
                                                            Lox P

        CTGTTGAAAAACCATACTTACGGATCTGGCTTCTGAGatggaccattgcaaaggactattgtgttct...
                                                      **********************
        cattatacgaagttatctcgagtcgctcgaattcctgcagcccgggtgcaaaggactattgtgttct...
```
