# Supplementary material for: Generation of a Mouse Model with Down-Regulated U50 snoRNA (SNORD50) Expression and Its Organ-Specific Phenotypic Modulation
Source: PLoS One. 2013 Aug 26;8(8):e72105. doi: 10.1371/journal.pone.0072105 (PMC3753356; doi:10.1371/journal.pone.0072105)
Supplement: Table S1 — Anomalies detected in age-matched wild-type and ΔmU50(HG-b) mice. Age-matched mice that were euthanized or had died were examined pathologically (n = 40 per genotype). (DOC) [file pone.0072105.s007.doc]

**Table S1.** Anomalies detected in age-matched wild-type and ΔmU50_(HG-b)_ mice

**ID Genotype Note Age (weeks)**

M160 ΔmU50 Euthanized swollen LN (inguinal) 98.0

F154 ΔmU50 Dead splenomegaly 93.6

F622 ΔmU50 Euthanized swollen LN (inguinal) / hind limb paralysis 89.4

F218 ΔmU50 Euthanized swollen LN (axillary and inguinal) 88.9

M223 ΔmU50 Euthanized swollen LN (axillary, mediastinal, mesenteric, and inguinal) 88.9

M221 ΔmU50 Euthanized - 88.7

F219 ΔmU50 Euthanized swollen LN (submandibular and inguinal) 87.6

F220 ΔmU50 Euthanized - 87.6

M503 ΔmU50 Euthanized - 84.0

F299 ΔmU50 Euthanized - 75.6

F300 ΔmU50 Euthanized - 75.6

F293 ΔmU50 Euthanized - 71.6

F295 ΔmU50 Euthanized - 71.6

F540 ΔmU50 Euthanized - 71.0

F541 ΔmU50 Euthanized - 71.0

F294 ΔmU50 Euthanized swollen LN (mesenteric) 70.6

M549 ΔmU50 Euthanized - 70.4

M551 ΔmU50 Euthanized - 70.4

M296 ΔmU50 Euthanized - 69.6

M222 ΔmU50 Euthanized pulmonary edema 68.0

F400 ΔmU50 Euthanized - 62.4

M367 ΔmU50 Euthanized - 60.4

M368 ΔmU50 Euthanized - 60.4

M369 ΔmU50 Euthanized - 60.4

F766 ΔmU50 Euthanized - 60.1

M390 ΔmU50 Euthanized - 59.9

F493 ΔmU50 Euthanized - 59.7

F494 ΔmU50 Euthanized splenomegaly / swollen LN (cervical, axillary, and inguinal) 59.7

M298 ΔmU50 Dead cardiac hypertrophy / pulmonary edema / splenomegaly 55.4

F724 ΔmU50 Euthanized - 53.6

M388 ΔmU50 Euthanized - 50.0

M297 ΔmU50 Dead cardiac hypertrophy / pulmonary edema 48.6

F387 ΔmU50 Euthanized - 48.3

F366 ΔmU50 Euthanized - 45.6

M550 ΔmU50 Euthanized - 45.4

F365 ΔmU50 Euthanized - 44.6

M591 ΔmU50 Euthanized - 41.1

F716 ΔmU50 Dead unknown cause 39.0

M389 ΔmU50 Dead lymphoid tumor / swollen LN (rectal) 36.0

F292 ΔmU50 Euthanized - 35.0

**Table S1.** Continued

**ID Genotype Note Age (weeks)**

F152 WT Euthanized swollen LN (inguinal and lumbar) / splenomegaly 98.6

F184 WT Euthanized - 92.1

M217 WT Euthanized - 89.9

M212 WT Euthanized - 89.7

F213 WT Euthanized - 88.6

M225 WT Dead unknown cause 83.6

M512 WT Euthanized - 82.4

F700 WT Euthanized - 80.6

F701 WT Euthanized - 80.6

F702 WT Euthanized - 80.6

F224 WT Dead splenomegaly / pulmonary edema 72.6

F302 WT Euthanized - 71.6

F303 WT Euthanized - 71.6

F301 WT Euthanized swollen LN (axillary and inguinal) 70.6

F554 WT Euthanized - 70.4

F555 WT Euthanized - 70.4

M557 WT Euthanized - 70.4

M558 WT Euthanized - 70.4

M306 WT Euthanized - 69.6

M307 WT Euthanized - 69.6

M713 WT Euthanized - 65.6

M764 WT Euthanized - 65.1

M376 WT Euthanized - 60.4

F377 WT Euthanized - 60.4

M384 WT Euthanized - 60.4

F553 WT Dead (dermatitis) 64.3

M374 WT Euthanized - 60.4

M375 WT Euthanized - 60.4

M792 WT Euthanized - 56.1

M382 WT Euthanized - 50.6

M383 WT Euthanized - 50.6

F380 WT Euthanized - 49.9

F378 WT Euthanized - 49.6

F730 WT Euthanized - 49.0

F379 WT Euthanized - 48.9

M757 WT Euthanized - 48.6

F373 WT Euthanized - 45.6

F372 WT Euthanized - 44.6

M308 WT Dead cardiac hypertrophy / pulmonary edema / congestive liver 36.0

F153 WT Euthanized - 35.0
