# Supplementary material for: Generation of a Mouse Model with Down-Regulated U50 snoRNA (SNORD50) Expression and Its Organ-Specific Phenotypic Modulation
Source: PLoS One. 2013 Aug 26;8(8):e72105. doi: 10.1371/journal.pone.0072105 (PMC3753356; doi:10.1371/journal.pone.0072105)
Supplement: Table S2 — List of PCR primers used in this study. (DOC) [file pone.0072105.s008.doc]

**Table S2. PCR primers used in this study.**

| **Primer name Sequence (5' to 3')** |
| --- |
| **Genotyping (*mU50HG-b*)** |
| B+0.6F GGTGTGTATTGAAGCCT |
| mR13 CAATCACTGCCAGAATAAGG |
|  |
| **Primer extension assay** |
| mU50_s1 TCTATGATGATCCTATCCCG |
| mU50_s2 TGATCCTATCCCGAACCTGA |
| mU50_as3 AAGCCAGATCCGTAATTATG |
| mU50_as4 ATCTCAGAAGCCAGATCCGT |
| mU50_1-20_A647 TCTATGATGATCCTATCCCG |
| mU50_70-51_FAM ATCTCAGAAGCCAGATCCGT |
| mU50_31-68_FAM CTCAGAAGCCAGATCCGTAATTATGGTTTTTCAACAGG |
| mU50_31-68_rv-cont_FAM GGACAACTTTTTGGTATTAATGCCTAGACCGAAGACTC |
|  |
| m_rDNA_2537s CCATATCCGCAGCAGGTCT |
| m_rDNA_2662as CGACCGACCCAGCCCTTA |
|  |
| **Real-time qPCR** |
| Bcl6_F TCGTGGAGAACAATATGCCA |
| Bcl6_R AAGAGGCTGGTGGTGTTGAC |
| c-Myc_F TAACTCGAGGAGGAGCTGGA |
| c-Myc_R AATTCCAGCGCATCAGTTCT |
| Lifr_F TGGTGCTCGCAGGCTCAAACA |
| Lifr_R TCGCAACGGTTTGCCCGTACA |
| Slc15a2_F AGCTACCCACTCAGCATCGCCT |
| Slc15a2_R AGCAGAGGCTGCTGAAGGCA |
| Tbc1d25_F AGGCCATGCCTTTGTATGCT |
| Tbc1d25_R TTGAGGTGGGCAAACTTGGT |
| Dvwa-Col6a4_F TCCACGCAGCGGACATTGCT |
| Dvwa-Col6a4_R TGCCTCTGGGAGGTTGTGCT |
| Xlr3a_F ACATTATATCAGCCGAGACCCGACC |
| Xlr3a_R GCCAGCAGTGTCAGTGGCCT |
| Dnaja1_F AGACTGCCTCCCCTGAACCTGA |
| Dnaja1_R AGTGGTGCCTGGTGCCAGTT |
| Hsph1_F CGCATGTGTGTGGCTGTGGT |
| Hsph1_R TGCTTCGCATGCTGCTACGGT |
| Hspa4l_F TCAGTGTGGCCAGTGCGTCA |
| Hspa4l_R AGCTTCCGTCTCCATAGCGGCA |
| Hspa1b_F TGATAGCTGCTTGGGCACCGA |
| Hspa1b_R TGCACAGTGCTGCTCCCAACA |
| Hspa1a_F TGGACTCTCCCCTGGGCCAC |
| Hspa1a_R CCAGCCCTGGCCTCTGAAGGA |
| Fscn1_F ACGGCAACGTGACCTGCGAG |
| Fscn1_R CGCCAAAGTAGCGCCGGTGA |
| Gm13051_F ACAGCCAGTACATTGTCCATGAACACA |
| Gm13051_R AGAATGACATTGCTAAGCTTGTCCCAT |
| mU50HGa_TOP_F1 GGACCATGGCTCATCATTCT |
| mU50HGa_TOP_R1 GAGTCAGGCTTTAATCAAGTTAT |
| mU50HGa_site1to3_F1 GGTAACATCCAGAAAAGGCAGAGACAA |
| mU50HGa_site1to3_R1 GACCCCATCTCAAACACCCCAGT |
| mU50HGb_F1 GGGTACTGGTGTAAACTTCC |
| mU50HGb_R1 GATGTCTCTTTCGGTGACTC |
